# Identification of the properties of H5 influenza vaccine viruses with high hemagglutinin yields

**DOI:** 10.1371/journal.pone.0280811

**Published:** 2023-01-20

**Authors:** Tomoko Arita, Yasushi Suzuki, Noriko Shimasaki, Hirotaka Kobayashi, Hideki Hasegawa, Takato Odagiri, Masato Tashiro, Eri Nobusawa

**Affiliations:** 1 Research Center for Influenza and Respiratory Viruses, National Institute of Infectious Diseases, Musashi-murayama, Tokyo, Japan; 2 Department of Virology III, National Institute of Infectious Diseases, Musashi-murayama, Tokyo, Japan; 3 Department of Pathology, National Institute of Infectious Diseases, Sinjuku, Tokyo, Japan; Foshan University, CHINA

## Abstract

Manufactured influenza vaccines have to contain a defined amount of hemagglutinin (HA) antigen. Therefore, vaccine viruses with a high HA antigen yield (HAY) are preferable for manufacturing vaccines, particularly vaccines in response to a pandemic, when vaccines need to be rapidly produced. However, the viral properties associated with a high HAY have not yet been fully clarified. To identify the HAY-associated traits, we first propagated 26 H5 candidate vaccine viruses (CVVs) in eggs, which were previously developed based on genetic reassortment methods using master viruses, to determine their total protein yield (TPY), ratio of HA to total viral protein (%-HA content) and HAY. The results revealed that the HAY was correlated with the TPY but not with the %-HA content. We further found that altering the sequences of the 3’ noncoding region of HA vRNA or replacing the master virus improved the HAYs and TPYs of the low-HAY CVVs to approximately double the values of the original CVVs but did not change the %-HA content, which a previous study suggested was associated with the HAY. Analyses based on real-time PCR assays and scanning electron microscopy revealed that the virus samples with an improved HAY contained more copies of the virus genome and viral particles than the original samples. The results suggest that an improvement in virus growth (i.e., an increase in the amount of viral particles) leads to an increase in the TPY and thus in the HAY, regardless of the %-HA content. The approximately twofold increase in the HAY shown in this study may not appear to represent a large improvement, but the impact will be significant given the millions of chicken eggs used to produce vaccines. These findings will be informative for developing high-HAY vaccine viruses.

## Introduction

Since the emergence of a highly pathogenic avian influenza (HPAI) A virus, A/goose/Guangdong/1/96 (Gs/GD) (H5N1), in China in 1996, the viruses of the Gs/GD lineage have continued to cause outbreaks in poultry in Asia, Europe, the Middle East and Africa, and sporadic human cases have also been reported. Due to the high mortality rate of humans infected with H5 HPAI viruses, outbreaks of the HPAI H5N1 and H5Nx viruses have been regarded as a great threat to our society since the first fatal case in 1997. Since the World Health Organization (WHO) declared a phase 3 pandemic outbreak of H5N1 influenza in 2004, WHO-affiliated laboratories worldwide have been developing H5N1 candidate vaccine viruses (CVVs), and new H5 CVVs continue to be developed in line with changes in antigenicity.

Influenza vaccines must meet at least two requirements: safety and immunogenicity. Regarding immunogenicity, the amount of hemagglutinin (HA) antigen in the vaccine has been considered an indicator of the immunogenic potency of the vaccine, and manufactured influenza vaccines must contain a defined amount of HA antigen when the conventional method for propagating vaccine viruses in eggs or cultured cells is used for inactivated vaccine production. Therefore, vaccine viruses with a high yield of HA antigen and high growth capacity are suitable for vaccine production, particularly those used in pandemic periods, when rapid production and sufficient supplies are needed. The HA antigen yield (HAY) of a vaccine virus is estimated from the total protein yield (TPY) and the percentage of HA protein relative to the total viral protein content (%-HA content).

Pandemic and prepandemic influenza vaccine viruses are generally developed as reassortant viruses carrying surface antigen (HA and neuraminidase (NA)) genes derived from a wild-type virus and internal genes from a master virus by either classical reassortment in eggs or reverse genetics (RG) technology. To develop a high-growth reassortant virus with an increased HAY in eggs, A/Puerto Rico/8/1934 (PR8), a well-known egg-adapted high-yield virus, has commonly been utilized as a master virus. However, due to the different passaging histories in each laboratory, all PR8 strains do not always exhibit the same growth properties. Johnson and colleagues previously investigated the influence of the internal genes derived from three different PR8 lineages on the HAYs of corresponding reassortant viruses [1], and these researchers found that no single internal PR8 gene set produced a higher HAY than others for all examined viruses but that a specific combination of a certain PR8 strain and individual HA/NA genes contributed to a high HAY. Conversely, their results also demonstrated a striking linkage between the HA/NA genes and HAY regardless of the internal PR8 genes [1].

Regarding H5N1 CVVs, an early H5N1 CVV, NIBRG-14, yielded a low amount of HA antigen and this finding has led to substantial efforts to improve and evaluate the HAYs of CVVs [1–6]. NIBRG-14 was created in response to an urgent request from the WHO in 2004; due to time constraints, the virus was generated using primers containing the consensus sequences of noncoding regions (NCRs) of H5N1 HAs and NAs instead of the sequences of VN1194, the parent virus of NIBRG-14 [7]. A subsequent analysis of mutant viruses originating from NIBRG-14 showed that mutations in the 3’NCR sequence of HA vRNA (3’NCR/HA) have little effect on the %-HA content and virus growth assessed by the hemagglutination assay [7].

A ‘high HAY’ is one of the essential properties of vaccine viruses, but the traits associated with the HAY have not yet been clearly identified. Previous studies have suggested that the inefficient incorporation of HA into the viral particles (i.e., a low %-HA content) was the reason for a low virus HAY [1, 3, 4, 7, 8]. However, these studies were performed by different laboratories under different conditions. In this study, to identify the properties associated with HAY, we first propagated 26 existing H5 CVVs in embryonated chicken eggs under the same conditions, characterized their growth and yields, and then evaluated their HAYs. Consequently, these 26 CVVs were classified into three groups, namely, the high-, intermediate- and low-HAY groups; however, the %-HA content was not necessarily correlated with the HAY. Therefore, to explore other properties related to the HAY, we attempted to improve the HAYs of the viruses in the low-HAY group by altering the 3’NCR/HA or replacing the master virus. The results suggested that these modifications of the viruses improved virus growth, which led to an increase in the TPY and thus an increase in the viral HAY but not the %-HA content.

## Materials and methods

### Viruses and cells

The H5 CVVs used in this study, as well as their designations, are shown in S1 Table. A human embryonic kidney cell line (HK293T) and Madin Darby canine kidney (MDCK) cells were cultured in DMEM (Thermo Fisher Scientific, Inc., MA, USA) and MEM (Thermo Fisher Scientific, Inc., MA, USA), respectively, supplemented with 10% fetal bovine serum. All cells were maintained under 5% CO_2_ at 37°C.

### Virus propagation and purification

Each CVV was propagated in six 10-day-old embryonated chicken eggs at 35°C. Ten milliliters of allantoic fluid was separately collected from each of the six eggs at 48 h post infection and separately subjected to HA titer determination. After confirming that the HA titers of the 6 samples were almost the same (if different, the difference was within 2-fold), the collected allantoic fluid was combined (total 60 ml) and clarified by centrifugation at 2,880 g (TOMY CAX-370) for 10 min at 4°C or 10,000 g (TOMY Suprema21) for 10 min at 4°C, if necessary. The supernatants were further purified by ultracentrifugation through 20% sucrose at 112,700 g for 90 min at 4°C using a Beckman SW28 rotor as described previously [9]. The pellets were resuspended in the appropriate volume of phosphate buffered saline (PBS) and then dialyzed with PBS overnight or ultracentrifuged at 112,700 g for 90 min at 4°C. The pellets were resuspended in 0.3 ml of PBS (viral concentrate). The purified dialyzed viruses were subjected to scanning electron microscopy. Each CVV was independently propagated three times and used in three independent experiments.

### Measurement of the total viral protein and HA protein levels

As described in previous studies [1, 3, 6, 8], we determined the total protein contents of the virus concentrates using a Micro BCA Protein Assay Kit (Thermo Fisher Scientific, Inc., MA, USA) with bovine serum albumin as a standard according to the manufacturer’s instructions. The TPY was calculated based on the total protein content and is presented as μg per ml of allantoic fluid and as μg per egg. The %-HA content of each virus was determined by SDS‒PAGE analysis as described previously [9–11]. In brief, samples were denatured at 100°C for 10 min and then treated with PNGase F at 37°C for 16 h according to the manufacturer’s instructions (New England Biolabs, MA, USA). The deglycosylated samples were boiled in Lane Maker Reducing Sample Buffer (Thermo Fisher Scientific, Inc., MA, USA) for 5 min at 95°C. A 4 or 8 μg sample was applied to each lane of a precast gel (12.5% e-PAGEL; ATTO). The levels of the HA1, HA2, NP, NA and M proteins were measured using the ATTO CS Analyzer 2.1 densitometry tool. The %-HA content (HA1 and HA2) was calculated as the percentage of total viral proteins composed of HA1, HA2, NP, NA and M. The HAY was estimated from the %-HA content and TPY and is reported as μg per ml of allantoic fluid and as μg per egg.

### Liquid chromatography‒tandem mass spectrometry (LC‒MS/MS) analysis of viral proteins

The identification of bands on SDS‒PAGE gels was performed by LC‒MS/MS using a modified method as described in a previous paper [12]. Briefly, differentiated bands were cut out and digested in gel with trypsin overnight. After digestion, peptides were extracted and analyzed using an LC‒MS/MS system consisting of a Chorur 220 Nano pump, PAL autosampler, nano spray, and Thermo LCQ DECA XP. The Thermo LCQ DECA XP was equipped with an LC of instrument in gradient elution mode. The gradient ran from 5% acetonitrile with 0.1% formic acid at 0 min to 95% acetonitrile with 0.1% formic acid at 20 min. The Thermo LCQ DECA XP detected MS/MS spectra via a trap mass spectrometer. The obtained MS/MS spectra were used in a search of a database based on the scoring probability using the search algorithm SEQUEST. The database was composed of peptides from whole influenza virus proteins of respective CVVs and fragments of chicken proteins and human proteins from EMBL-EBI data resources and tools (https://www.ebi.ac.uk). Chicken proteins such as actin and human proteins such as keratin were used as peptides that could have been accidentally carried over from eggs and by contamination from workers.

### Statistical analysis

The statistical analyses were performed with R software, version 4.1.1 [13]. The Pearson correlation coefficients between the data were calculated. Significant differences in the data were assessed with Tukey’s honest significant difference (Tukey’s HSD) method.

### Sequence analysis of virus genomes

Total RNA of each virus was extracted using a QIAamp viral RNA mini kit (Qiagen Hilden Germany) and reverse transcribed, and the cDNAs were amplified with a PrimeScript RT‒PCR kit (Takara Bio Inc. Shiga, Japan). The RT‒PCR products purified using NucleoSpin Gel and PCR Clean-up (Takara Bio Inc. Shiga, Japan) were subjected to sequence analysis using a Big Dye Terminator v3.1 cycle sequencing kit (Thermo Fisher Scientific, Inc. MA, USA) and a 3130 Genetic Analyzer (Thermo Fisher Scientific, Inc. MA, USA). The sequences of the primers used for RT‒PCR and the sequence analysis data are available.

### Sequencing of 5’ and 3’NCRs of HA and NA genes

Using an RNeasy Mini kit (Qiagen Hilden Germany), total RNA was extracted from the virus concentrates. To accurately determine the sequences of the 5’ and 3’ NCRs of the HA and NA viral RNAs (vRNAs), we performed rapid amplification of cDNA ends (RACE) with the extracted RNA using a SMARTer RACE 5’/3’ kit according to the manufacturer’s instructions (Takara Bio Inc. Shiga, Japan). To determine the sequences of the 5’ and 3’NCRs, cDNA was synthesized separately for each end using appropriate primers for RACE (included in the kit) and gene-specific primers (S2 Table). Before the 3’RACE reaction, a poly(A) tail was added to the 3’ end of the vRNA using a Poly(A) Polymerase (Takara Bio Inc. Shiga, Japan) according to the manufacturer’s instructions. The RACE fragments were gel purified with a NucleoSpin Gel and PCR Clean-Up kit (Takara Bio Inc. Shiga, Japan), and the purified products were subjected to sequence analysis.

### Generation of reassortant viruses by RG technology

Reassortant viruses were generated using two different RG systems: a 12-plasmid RG system [14] and an 8-plasmid RG system [15]. The plasmids of each system encoded viral genes of two different PR8 lineages, namely, PR8-a and PR8-b. Differences in the amino acid sequences of these viral proteins encoded by the respective internal genes are provided in S3 Table. Various amino acid differences were observed in PB2, PB1, PB1-F2, PA, PA-X, NP, M2, NS1 and NS2 but not in M1. The differences are almost consistent with those reported by Johnson and colleagues [1], and PR8-a and PR8-b appear to correspond to Backbone-B (PR8-B) and Backbone-A (PR8-A) from their study, respectively. The HA and NA genes were amplified from the virus stock by reverse transcription with SuperScript II Reverse Transcriptase (Thermo Fisher Scientific, Inc., MA, USA) and PCR with KOD-Plus-Neo (TOYOBO CO., LTD., Osaka, Japan) using primers specific to the 5’ and 3’NCRs of each gene (S2 Table). The PCR products of the HA and NA genes were subcloned into the RG plasmid vectors pHH21 (12 plasmid system) [14] and pCIPolISapIT (8 plasmid system) [15], which are under control of the human RNA polymerase I promoter and the mouse RNA polymerase I terminator. For the 12-plasmid RG system, the pHH21 plasmids encoding HA and NA genes of H5N1 viruses, six internal genes of PR8-a and four pCAGGS plasmids encoding PB2, PB1, PA and NP proteins of PR8-a were cotransfected into HK 293T cells. For the 8-plasmid RG system, pCIPolISapIT encoding HA and NA genes of H5N1 viruses and six internal genes of PR8-b was cotransfected into HK 293T cells. Transfection was performed using Trans^®^IT-LT1 Transfection Reagent (Mirus Bio LLC. WI, USA) according to the manufacturer’s instructions. At 48 h post transfection, the supernatant was collected and inoculated into MDCK cells, and the cells were cultured at 37°C for 48 h in infection medium containing 10 μg/ml acetylated trypsin (Sigma‒Aldrich Co., LLC, MO, USA) according to a previous study [9, 11]. The generated viruses were propagated in 10-day-old embryonated chicken eggs at 35°C for 48 h. We have confirmed that all the resulting RG viruses had no unexpected mutations in the HA and NA genes, including in the NCRs and coding regions.

### Real-time PCR

The reactions were performed using AgPath-ID™ One-Step RT‒PCR Reagents (Thermo Fisher Scientific, Inc., MA, USA) with a LightCycler® 480 II (Roche, Basel, Switzerland). The final reaction volume of 25 μl contained 12.5 μl of 2 × RT‒PCR buffer, 1 μl of 25 × RT‒PCR enzyme mix, 0.1 μl (20 U) of RNase inhibitor (Thermo Fisher Scientific, Inc., MA, USA), 600 nM each forward and reverse primer, 100 nM TaqMan MGB probe and 5 μl of vRNA. vRNA was extracted from infected allantoic fluid or viral concentrates using an RNeasy Mini kit (Qiagen Hilden Germany). Prior to use, infected allantoic fluid was treated with RNase to remove free-floating RNA (not associated with viral particles), but no difference in the results was found between the RNase-treated and untreated samples. The copy numbers of each vRNA are reported per ml of the allantoic fluid and the values between the viruses were then compared. Information on the primers and probes is provided within the WHO information on the molecular diagnosis of influenza viruses (https://www.who.int/teams/global-influenza-programme/laboratory-network/quality-assurance/eqa-project/information-for-molecular-diagnosis-of-influenza-virus). The cycling conditions were as follows: 50°C for 10 min, 95°C for 10 min, and 45 cycles of 15 s at 95°C, 30 s at 56°C and 15 s at 72°C. Amplification data were collected at 56°C (annealing step) and analyzed using the second derivative maximum method with LightCycler® 480 SW1.5 software.

### Scanning electron microscopy (SEM) analysis

A fraction of the dialyzed purified viruses was fixed with 2.5% glutaraldehyde/2% paraformaldehyde overnight at 4°C and placed on a cover glass. The cover glasses were treated with 1% osmium tetraoxide at room temperature for 30 min. The samples were dehydrated through a graded acetone series (50%-100%) at room temperature and subjected to critical point drying and osmium plasma coating using a critical point dryer (CPD300, Leica) and an osmium plasma coater (Neoc-Pro/P, Meiwafosis). Images were obtained with a scanning electron microscope (Regulus8220, Hitachi High-Tech). The numbers and diameters (nm) of the viral particles were measured using Image-Pro (Version 10.0.2). The measurements were exported to Microsoft Excel to calculate the number and proportion of each particle based on the area as follows. For each sample, we randomly selected 20 fields of view, determined the number of particles within the specified particle size range (3000nm^2^~8000nm^2^) for each field of view, and confirmed that the number of particles was normally distributed by the Shapiro‒Wilk test using GraphPad Prism software. We then verified that the variation in the number of particles in each field of view was not outside of the normal distribution using the Smirnov-Grubbs test. Statistical significance was set to P < 0.05.

## Results

### Growth characteristics of CVVs

**Fig 1:**
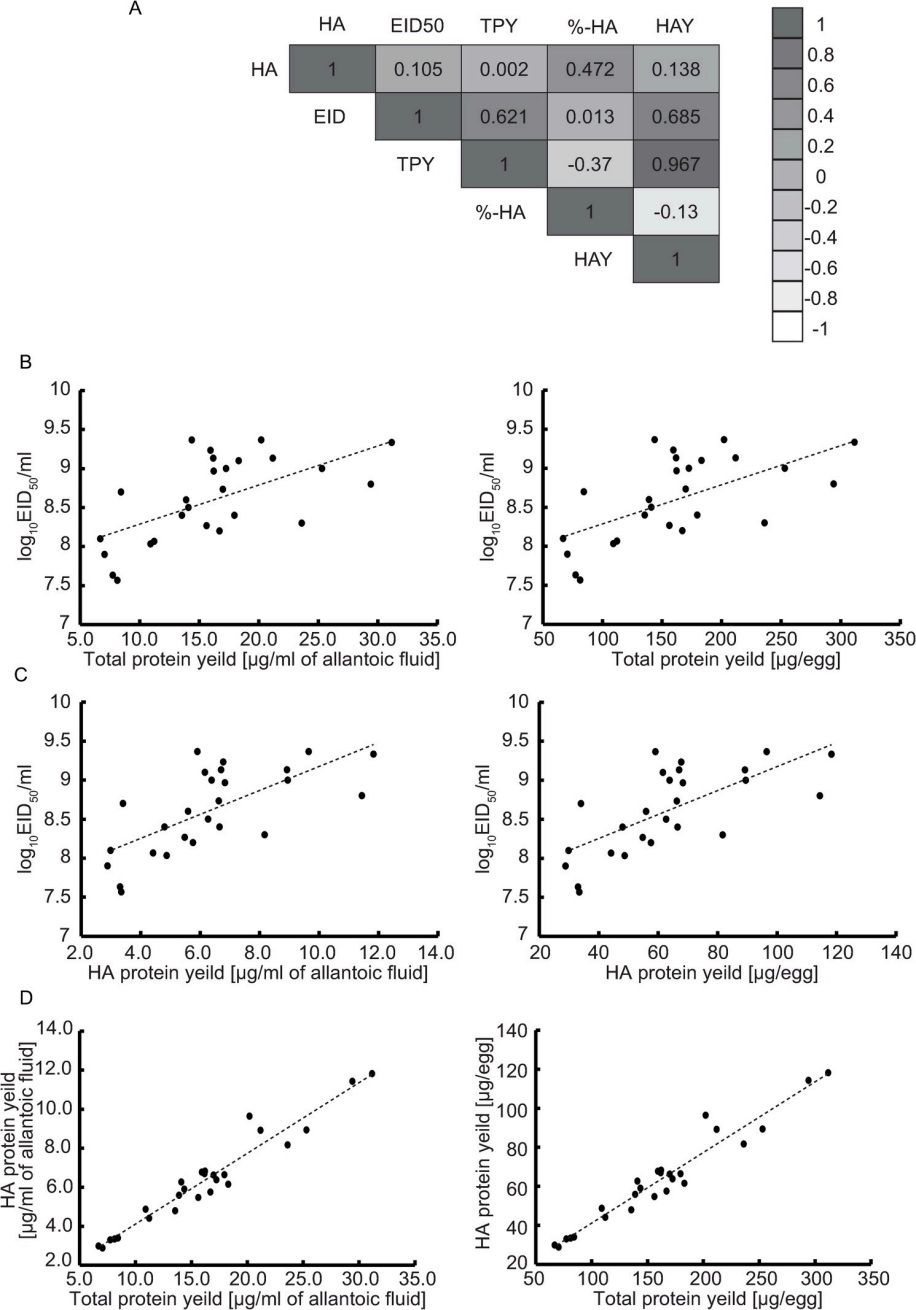
Correlations between different virus characteristics. The Pearson correlation coefficients among viral characteristics were calculated using R version 4.1.1 software^13)^. (A) The correlation matrix of virus characteristics is shown. The abbreviations of each characteristic are as follows: HA titer (HA), 50% Egg Infectious Dose (EID50), total protein yield (TPY), %-HA content (%-HA), HA antigen yield (HAY). (B) Correlations between the EID50 and TPY of 26 H5 CVVs. (C) Correlations between the EID50 and HAY of 26 H5 CVVs. (D) Correlations between the HAY and TPY of 26 H5 CVVs.

To evaluate the growth properties of 26 CVVs in eggs, we first measured HA titer and 50% egg infectious dose (EID_50_) of each CVV three times, independently (S4 Table). The average EID_50_ titers ranged from 10^7.6^ to 10^9.4^ EID_50_/ml, whereas the HA titers ranged from 2^7.0^ to 2^11.0^. As shown in Fig 1A and S4 Table, the HA titers were not always correlated with the infectivity titers (EID_50_/ml), for example, three CVVs (N, V, and Y) showed the average HA titers of 2^8.0^~2^8.3^, and the other three CVVs (F, J, and M) exhibited mean HA titers of 2^9.7^~2^10.0^, while the mean EID_50_ values of the former CVVs (N, V, and Y) ranged from 10^9.0^ to 10^9.4^EID_50_ and the mean EID_50_ values of the latter CVVs (F, J, and M) were 10^7.6^~ 10^8.0^.

### Evaluation of the TPYs of the CVVs

The TPY of each CVV was measured using the viral concentrates as described in the Materials and Methods. The average values of each TPY showed a wide range from 6 to 31 μg/ml (60 to 310 μg/egg) (Fig 1 and S4 Table). As shown in S4 Table, F-, G-, I- and J-CVV showed similar or lower TPY values than A-CVV (8.4 μg/ml, 84.2 μg/egg), while the remaining CVVs showed higher TPY values than A-CVV. Among these CVVs, fifteen CVVs exhibited TPY values almost double or more than twice that of A-CVV. The comparison of the TPY values with the EID_50_ titers and HA titers revealed that the TPY exhibited a weak correlation with the EID_50_ titers but not the HA titers (Fig 1A and 1B).

### SDS‒PAGE analysis of the CVVs

**Fig 2:**
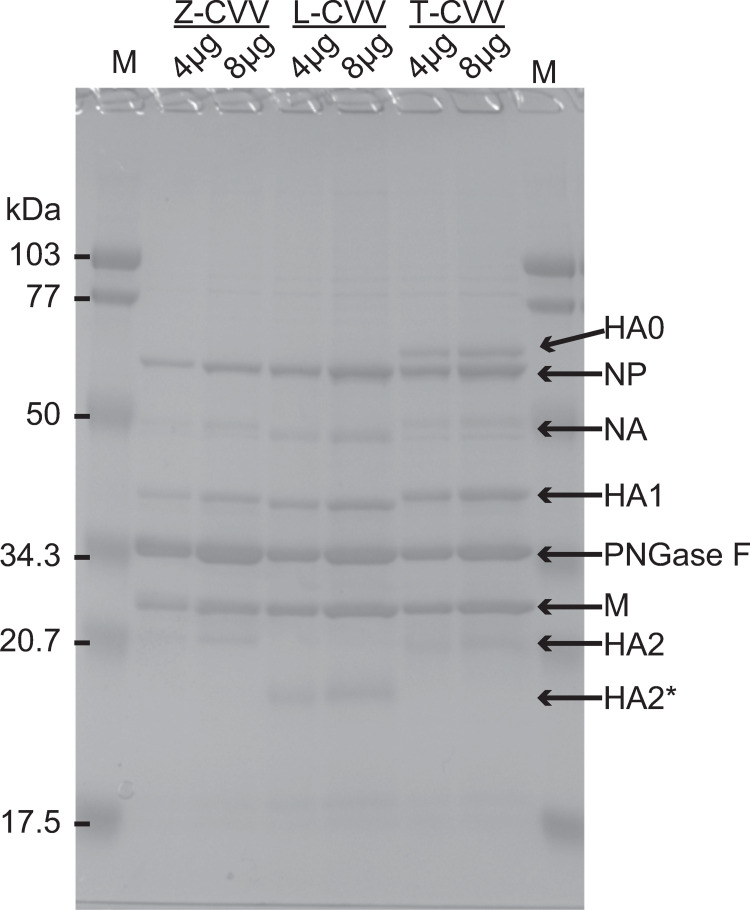
SDS‒PAGE analysis of viral concentrates of CVVs. SDS‒PAGE analysis of Z-CVV, L-CVV, and T-CVV. HA2* represents an HA2 with slower mobility than that of other CVVs. M indicates size markers.

To determine the %-HA contents, we analyzed the virus concentrate of each CVV by SDS‒PAGE analysis as described in the Materials and Methods. The deglycosylated HA1 and HA2 proteins of most CVVs migrated similarly and were distinguishable from other viral proteins (Fig 2 and S1 Fig). The migration pattern of Z-CVV was identified as a representative normal migration pattern (Fig 2). In contrast, the HA proteins of four CVVs (J, K, L, and T-CVV) showed faster or slower mobility than those of other CVVs (Fig 2 and S1 Fig). Previous studies have suggested that the differences in the sizes of HA proteins might reflect the differences in their amino acid sequences and/or levels of glycosylation. It has been reported that certain types of glycans that are not recognized by PNGaseF may affect the migration of each band [16, 17]. To confirm which band corresponded to which viral protein, we performed a LC‒MS/MS analysis of the bands exhibiting unexpected mobility. Hence, the smaller bands (approximately 29 kD) of the J-, K-, and L-CVV samples were identified as HA2, and the larger band (approximately 70 kD) of the T-CVV sample was identified as HA0 (Fig 2 and S1 Fig). We estimated the viral protein profiles of each CVV based on the density of each band including the bands (HA0 and HA2) exhibiting unexpected mobility. The average %-HA content of each CVV ranged from approximately 33% to 48% (S4 Table).

### HAYs of CVVs

The HAY of each CVV was estimated as described in the Materials and Methods. The HAYs ranged from approximately 3 to 12 μg/ml, 30 to 120 μg/egg, and the CVVs were classified into three groups: low-HAY (<4 μg/ml, <40 μg/egg), intermediate-HAY (4–8 μg/ml, 40–80 μg/egg) and high-HAY (>8 μg/ml, >80 μg/egg) (Fig 1C and S4 Table). The HAYs of the F-, G-, I- and J-CVVs were as low as that of A-CVV (3.4 μg/ml, 34.0 μg/egg), and the TPYs of these viruses were also as low as that of A-CVV. Overall, a good correlation was detected between the HAY and TPY of each CVV (Fig 1D): a higher TPY was associated with a higher HAY, regardless of the %-HA content. Each HAY value was weakly correlated with the EID_50_ titer (Fig 1C), and the same findings were observed for each TPY value (Fig 1B). In contrast, neither the TPY nor the HAY was necessarily correlated with the HA titer or %-HA content (Fig 1A).

### Influence of 3’NCR sequences on the HAY

**Fig 3:**
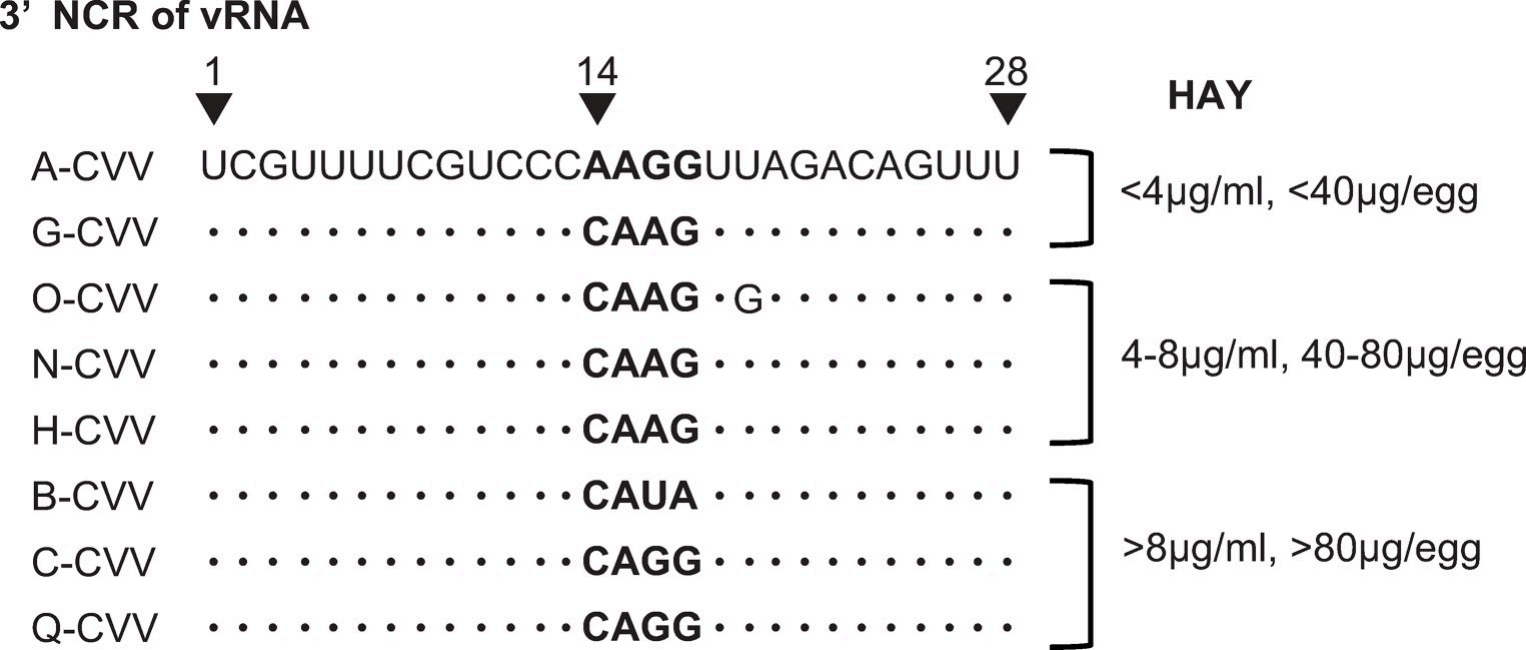
Nucleotide sequences of the 3’NCRs of HA vRNAs. Nucleotide sequences of the 3’ ends (1–28) of the HA vRNAs (negative sense) of each CVV. The nucleotide numbering starts from the 1^st^ nucleotide of the 3’NCR. The approximate HA yield (HAY) of each CVV is shown as μg per ml of allantoic fluid and μg per egg according to the values shown in S4 Table.

To explore the features that improve HAYs without affecting the antigenicity or function of HA proteins, we first focused on the 3’NCR/HA. To examine the effect of 3’NCR/HA on the HAY, we determined the sequences of the NCRs of HA and NA genes of the A-, B-, C-, G-, H-, N-, O-, and Q-CVVs (Fig 3 and S2 Fig). These eight viruses were randomly selected from the low-to-high HAY groups (S4 Table). The results showed that the NCR sequences at the 5’end of the HA gene and the 3’ and 5’ ends of the NA gene were almost identical among the eight CVVs (S2 Fig). However, four different sequences were observed at positions 14 to 17 of the 3’NCR of the HA gene (14-17/3’NCR/HA) (Fig 3). According to a previous study, ‘CAAG’ within the 14-17/3’NCR/HA sequences of G- CVV, H- CVV, O- CVV and N-CVV was identical to that of A/Vietnam/1194/2004 (VN1194), a parent virus of A-CVV [7].

**Fig 4:**
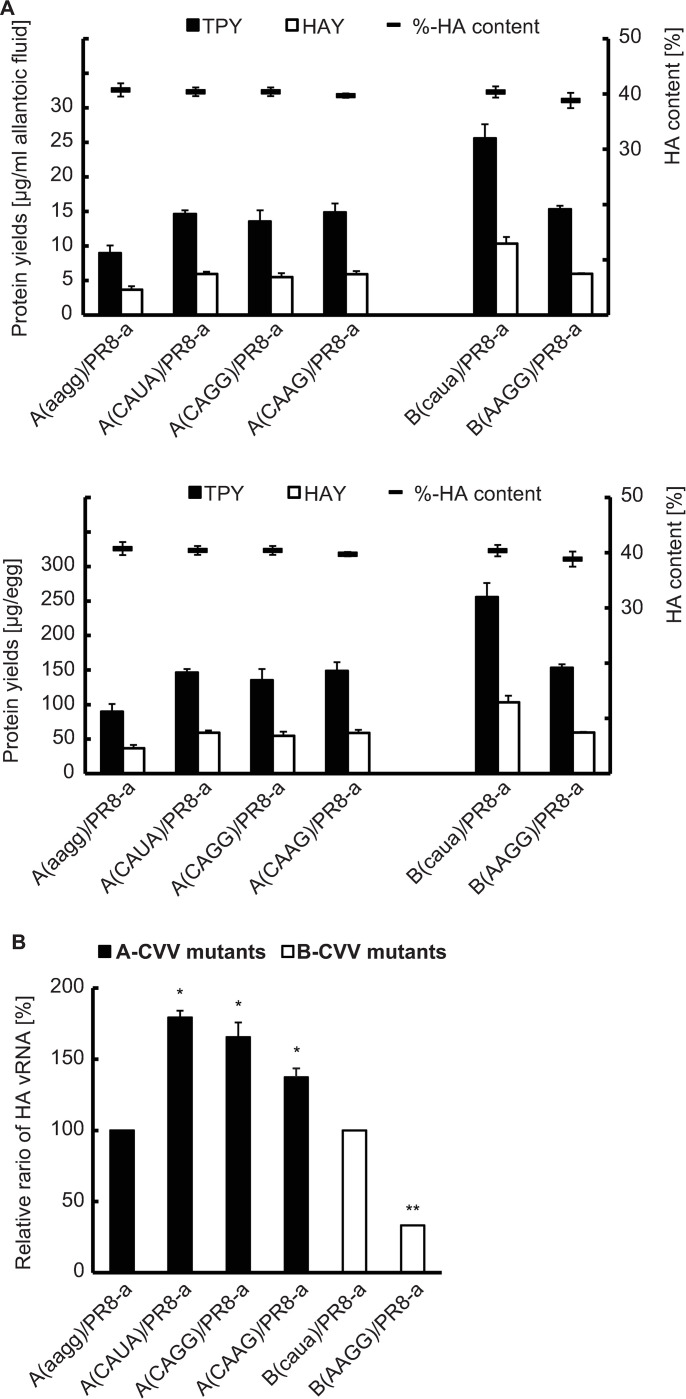
Characterization of 3’NCR/HA mutants. (A) Average total protein yield (TPY), average HA antigen yield (HAY) and average %-HA content of each mutant. (B) Relative HA vRNA ratio of each mutant. The vertical axis indicates the ratios of vRNAs to A(aagg)/PR8-a and B(caua)/PR8-a. A single asterisk and double asterisks indicate significant differences (*P* < 0.01) from the value of A(aagg)/PR8-a and the value of B(caua)/PR8-a, respectively. The differences in the TPY, HAY, %-HA content and the relative HA vRNA ratio were statistically analyzed using Tukey’s HSD method. The values are presented as the mean values with standard deviations from three independent experiments.

A previous study found that changes in the 14-17/3’NCR/HA sequence of A-CVV had a minimal impact on the HA titers, infectivity titers and %-HA contents of the viruses, but the corresponding HA yield values were not given [7]. To examine the effect on the HAY, we generated three 3’NCR/HA mutants carrying different sequences, ‘CAUA’, ‘CAGG’, and ‘CAAG’ within 14-17/3’NCR/HA of A-CVV (S5 Table). These sequences are the same sequences whose influence on the growth and %-HA content of viruses was examined in the aforementioned study [7]. To avoid the effect of the master virus on the results, we first replaced the master virus of A-CVV with PR8-a and confirmed that the infectivity titers, TPY, %-HA content and HAY of A(aagg)/PR8-a were comparable to those of A-CVV. However, comparatively lower HA titers were observed with A(aagg)/PR8-a compared to A-CVV (S6 Table). The analysis of the three mutants, A(CAUA)/PR8-a, A(CAGG)/PR8-a, and A(CAAG)/PR8-a, showed that the HA titers, infectivity titers and %-HA content of these mutants were similar to those of A(aagg)/PR8-a, as demonstrated in a previous study [7], whereas the TPYs and HAYs of these mutants were approximately 1.5~1.6 fold higher than those of A(aagg)/PR8-a (Fig 4A and S6 Table). On the other hand, all the RG viruses exhibited comparatively lower HA titers than A-CVV. The only common factor among the RG viruses was the master virus PR8-a, but to clarify the reason for the low HA titers, further investigation is needed.

Among the eight CVVs shown in Fig 3, the A- and B-CVVs belonged to clade 1 and were isolated at similar time points, and the HAY of B-CVV was approximately three times higher than that of A-CVV (S1 and S4 Tables). The 14-17/3’NCR/HA nucleotide sequence of B-CVV was CAUA. Therefore, to investigate the influence of AAGG in 14-17/3’NCR/HA on the TPY and HAY of B-CVV, we created B(AAGG)/PR8-a and examined the effect (S5 Table). The results revealed the TPY and HAY of B(AAGG)/PR8-a was approximately 60% of those of B(caua)/PR8-a (Fig 4A and S6 Table). To avoid the influence of the master virus on the effect, we again created B(caua)/PR8-a and confirmed that the TPY and HAY values of B-CVV and B(caua)/PR8-a were similar (S5 and S6 Tables).

To further investigate whether the 14-17/3’NCR/HA sequence solely affected the HAY regardless of the coding region of the HA gene, we generated two additional mutant viruses, (AAGG)/PR8-a and (CAUA)/PR8-a (S5 Table). Eight genome segments of both viruses were derived from those of PR8-a, although the NCRs of the HA and NA segments were not. Both NCRs were derived from those of A-CVV, with differences at the 14-17/3’NCR/HA positions (S5 Table). As a result, the TPY and HAY of (CAUA)/PR8-a were approximately 1.6~1.7 times higher than those of (AAGG)/PR8-a (S6 Table). Again, no significant differences in the %-HA content were observed between (AAGG)/PR8-a and (CAUA)/PR8-a. These results suggested that the 14-17/3’NCR/HA nucleotide sequences might affect the TPY and HAY regardless of the sequences in the HA coding region. The 14-17/3’NCR/HA sequences were likely correlated with the HAY.

To determine how the 14-17/3’NCR/HA sequences affected the TPYs and HAYs of the viruses without causing differences in the %-HA values, we focused on virus growth. For quantification of virus growth, we adopted a method that is independent of the viral receptor-binding ability and infectivity. We measured the HA vRNA contents of the samples of 3’NCR/HA mutants derived from A-CVV and B-CVV using a real-time PCR assay as described in the Materials and Methods. The estimated HA vRNA levels of the A-CVV mutants A(CAUA)/PR8-a, A(CAGG) /PR8-a and A(CAAG)/PR8-a were approximately 1.8~1.5 times higher than that of A(aagg)/PR8-a, whereas that of B(AAGG)/PR8-a was approximately 33% that of B(caua)/PR8-a (Fig 4B). These rates of change almost coincide with the rates of change found for the TPY and HAY of the corresponding mutants. The results implied that the changes in the 14-17/3’NCR/HA sequence affected the TPYs and HAYs through the changes in the HA vRNA content of the virus sample.

### Influence of the master virus on the HAYs

To identify other features correlated with the HAY, we attempted to improve the HAY of another low-HAY group virus, G-CVV. Although G-CVV and H-CVV belong to the same clade (clade 2.2) and their HA and NA amino acid sequences are almost the same (S1 and S7 Tables), the TPY and HAY (17.0 μg/ml, 169.8 μg/egg, and 6.6 μg/ml, 66.2 μg/egg, respectively) of H-CVV were more than two times higher than those of G-CVV (6.7 μg/ml, 66.8 μg/egg, and 3.0 μg/ml, 29.9 μg/egg, respectively) (S4 Table). The 14-17/3’NCR/HA sequence of both CVVs is CAAG. A previous study suggested that the low HAY of a certain CVV was due to the HA and/or NA genes themselves and not to a master virus [18]. To clarify whether the reason for the low HAY of G-CVV was the HA/NA genes or the master virus, we first generated reassortant viruses possessing the HA and NA genes of G-CVV and internal genes of PR8-a or PR8-b (G/PR8-a, G/PR8-b). No significant difference in the HA titer or infectivity titer was found among G-CVV, G/PR8-a and G/PR8-b (S6 Table). In contrast, the TPYs and HAYs of G/PR8-a (14.5 μg/ml, 145.3 μg/egg, and 5.9 μg/ml, 58.9 μg/egg, respectively) and G/PR8-b (16.6 μg/ml, 166.2 μg/egg, and 6.3 μg/ml, 63.2 μg/egg, respectively) were twofold higher than those of G-CVV, whereas no significant difference in the %-HA contents was identified among these viruses, as observed with the 3’NCR/HA mutants (S6 Table). On the other hand, a slight difference was observed in the yield of G/PR8-a and G/PR8-b. It is possible that this difference was related to the dissimilarity in the amino acid sequences of the viral proteins encoded by the internal genes (S3 Table), but further investigation is needed to verify this hypothesis. Although we did not examine whether the difference in the amino acid sequences of HA and NA between G- and H-CVV affected the TPY or HAY, the results suggested that the low HAY of G-CVV might be attributed to the characteristics of the master virus. A similar influence of a master virus on the HAYs was reported by Johnson and colleagues [1]. However, further work is needed to determine whether or not the difference was due to the master virus or the differences in the sequences of HA and NA (or both).

**Fig 5:**
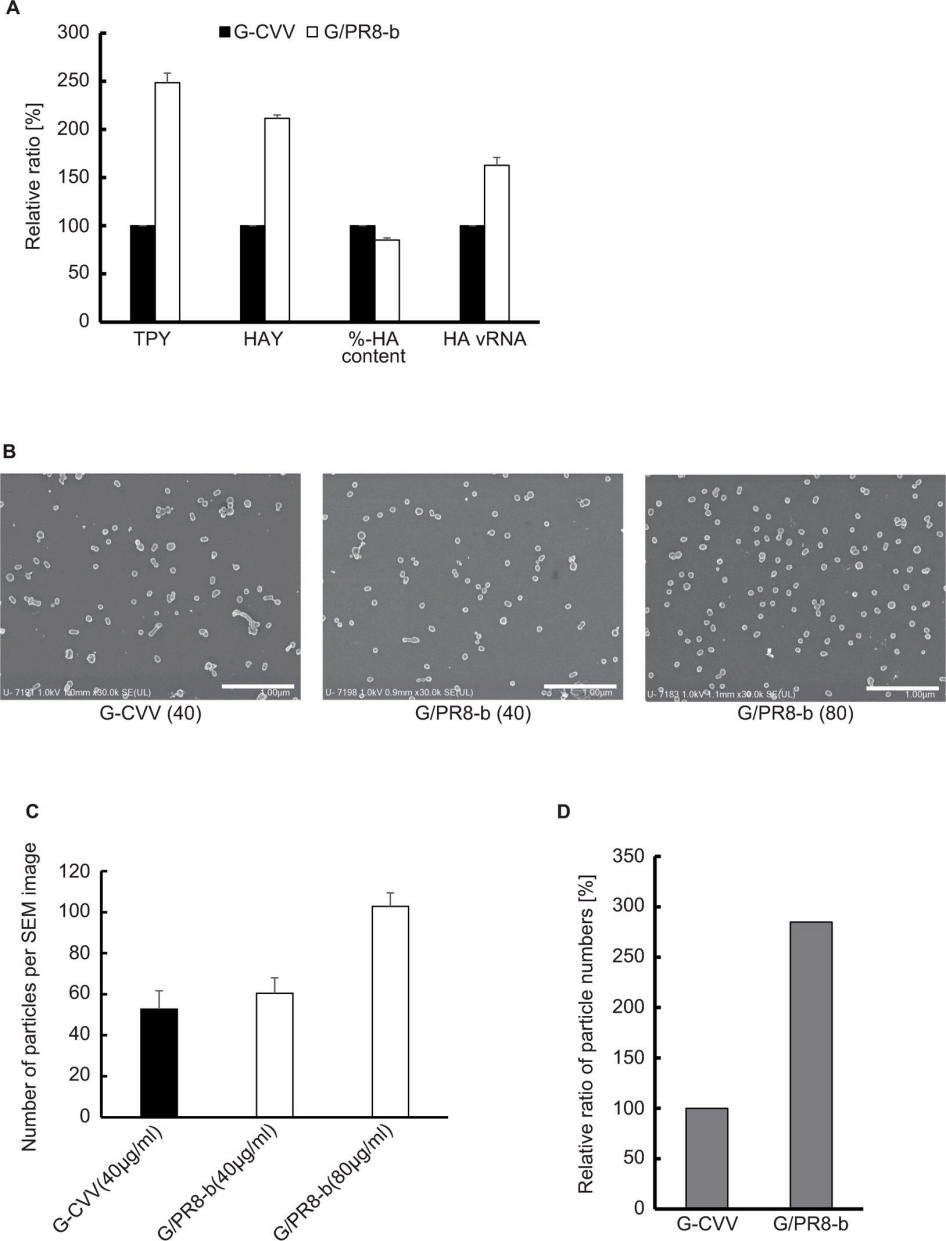
Characterization of master virus mutants. (A) Relative ratios of the total protein yield (TPY), HA antigen yield (HAY), %-HA content, and HA vRNA content between G-CVV and G/PR8-b. The values are presented as the mean values with standard deviations from three independent experiments. (B) Scanning electron micrographs of viral particles. Representative electron micrographs of G-CVV and G/PR8-b are shown. The protein concentrations of each sample, 40 μg/ml and 80 μg/ml, are shown as (40) and (80). The scale bars indicate 1 μm. (C) Average number of particles per field of view obtained for G-CVV and G/PR8-b. Average numbers were calculated from 20 fields of view. (D) Relative ratio of viral particle numbers in allantoic fluid obtained for G-CVV and G/PR8-b. The values are presented as the mean values with standard deviations from three independent experiments.

G/PR8-a and G/PR8-b both showed higher TPY and HAY values than G-CVV. To determine how the master virus affected the HAY, further analysis was performed with G/PR8-b and G-CVV. Similar to the analysis of the 3’NCR/HA mutants, we first compared the HA vRNA contents of the G-CVV and G/PR8-b samples. The estimated HA vRNA content of G/PR8-b was approximately 1.6 times higher than that of G-CVV, which suggested that alterations in the master virus affected the HA vRNA content of the virus sample, as observed with the 3’NCR/HA mutants (Fig 5A). An increase in the number of virus genome copies in the sample may be correlated with the increases in the TPY and HAY (Fig 5A).

The association between an increase in the genome copy number and an increase in the TPY suggests that the increase in the viral particle numbers resulted in an increased TPY. To clarify the correlation between viral particle numbers and protein content, we compared the viral particle numbers in the G-CVV and G/PR8-b samples by SEM. The samples were appropriately diluted to a protein concentration of 40 μg/ml and/or 80 μg/ml and subjected to SEM. The comparison of the viral particle numbers per field of view (adjusted to a protein concentration of 40 μg/ml) between G-CVV and G/PR8-b showed that the number of the particles was almost the same, whereas the viral particle numbers of G/PR8-b adjusted to protein concentrations of 40 μg/ml and 80 μg/ml showed a twofold difference depending on the protein concentration (Fig 5B and 5C). Based on the relationship between the protein concentration of each virus and the corresponding number of viral particles per field of view, we deduced the particle numbers per field of view in each allantoic fluid sample as follows: average particle number in one field at a protein concentration of 1 μg/ml multiplied by the TPY (μg/ml of allantoic fluid) of each sample (Fig 5D). The results showed that the number of viral particles in the G/PR8-b sample was more than twofold that in the G-CVV sample. These results suggested that the increase in the viral particle numbers was correlated with the protein content, which may have led to the improvement in the HAY.

As shown in S3 Fig, the comparison of the areas of the particles of both viruses showed that the G-CVV sample contained several larger and nonspherical particles, whereas the G/PR8-b sample had more-uniform spherical particles. However, since we examined the shape of virus particles in only 20 fields, further investigation is needed to determine the relationship between viral shape and TPY.

## Discussion

The experience gained from the ongoing COVID-19 pandemic and the influenza pandemic of 2009 firmly demonstrated that rapid vaccine production and sufficient vaccine supplies are important for mitigating the paralysis of social functions and damage to the health of individuals during a pandemic. During the 2009 influenza pandemic caused by the A/H1N1pdm09 virus, vaccine virus development began immediately after the outbreak of the pandemic using classical reassortment or reverse genetics technology. However, the HAYs of the A/H1N1pdm09 CVVs were estimated to equal 30–50% of those obtained with seasonal CVVs [19]. Because the influenza vaccine is manufactured with consideration of the HA antigen levels, vaccine viruses exhibiting a high HAY are desirable for vaccine production. However, previous studies have shown that the viral properties associated with the HAY are virus-specific and varied [1]. When a pandemic strikes, the rapid development of high-yielding CVVs is therefore not necessarily easy, and it is desirable to preestablish a reliable indicator that can quantitatively estimate the HAY level of vaccine viruses.

To date, numerous H5 CVVs have been developed by several different laboratories due to the possibility that the next influenza pandemic will be caused by an H5 HPAI virus. However, the HAYs of these viruses have not always been measured using the same method. In this study, to identify the viral properties related to the HAYs, we first determined the HAYs of 26 existing H5 CVVs using a single method and found that the HA titer and %-HA content showed little correlation with the HAY, whereas the TPY was correlated with the HAY (Fig 1). The infectivity titer exhibited weak correlation with the HAY. A previous study suggested that increase in HA incorporation into viral particles increases the %-HA content, which may in turn improve the HAY in some CVVs [1, 3, 18], but this finding was not obtained for several CVVs examined in this study. Some CVVs (such as G- and J-CVV) with %-HA content above 40% exhibited low TPYs and HAYs. Conversely, several CVVs (Q- and R-CVV) showed high TPYs and HAYs even when the %-HA content was less than 40% (S4 Table). Our work and some previous studies imply that improvements in the HAY are not necessarily caused by increases in the %-HA contents [1, 11, 20]. Among the methods used to estimate the HAY, isotope dilution mass spectrometry (IDMS) can be employed as an alternative approach. However, because Johnson and colleagues demonstrated that the HAY values determined by IDMS and SDS‒PAGE /densitometry are similar [1], we did not utilize IDMS in this study.

To improve the HAYs of low-HAY viruses, we attempted to change the 14-17/3’NCR/HA sequences of the viruses and to replace the master virus. For 3’NCR/HA, previous studies have suggested that these sequences are involved in selective viral genome packaging but do not impact the %-HA content [7, 21, 22]. Although the results did not show a direct effect on the HAY, our study revealed that the 14-17/3’NCR/HA sequence impacted the HAY but not the %-HA content. Moreover, this effect of the 14-17/3’NCR/HA sequence was not affected by the sequence of the coding region of the HA gene. Among our 3’NCR/HA mutants, all high-yield mutants showed CA at positions 14–15 of 3’NCR/HA (S5 and S6 Tables). We did not directly examine the influence of an A residue at position 15 (A15) on the HAY. According to a previous study, although A15 is conserved among 374 H5NCR sequences, it does not appear to be needed for viral replication [21–23]. Only the C nucleotide residue at position 14 may be relevant for viral replication and possibly also for the HAY.

An earlier study found that a change in the packaging signal of the NA gene could increase the viral growth titer and %-HA content [5]. Regarding the effect of the NCR of the NA gene (NCR/NA), the sequences of A-CVV (low-HAY) and B-CVV (high-HAY) were identical except for a single-base insertion in the A-CVV 5’NCR/NA (S2 Fig). To determine the effect of NCR/NA sequences on the HAY, we generated a modified version of A(CAUA)/PR8-a in which the NCR/NA sequence was changed to that of B-CVV (A(CAUA)B(NA NCR)/PR8-a), but little increase in the TPY and HAY values was detected (S5 and S6 Tables). Because we only changed the sequence of the NCR in the packaging signal region of the NA gene, we cannot exclude the possibility that changing the entire packaging signal sequence would have an effect.

To improve the virus growth and HAYs of CVVs, various master viruses have been developed by modifying or exchanging the internal genes [2, 4, 24, 25]. The genetic and functional compatibility between the HA/NA genes and the internal genes of a master virus is expected to be one of the factors influencing the HAY; however, identifying a proper combination of HA/NA genes and a master virus is not easy. Therefore, Johnson and colleagues proposed that developing CVVs using multiple master viruses, if available, and selecting a high-yield CVV among them would enable rapid vaccine development in a pandemic [1]. In our study, the HAY of G-CVV was improved by changing the master virus. However, in contrast to the results of some previous studies, no significant difference in the %-HA contents was detected between G-CVV and G/PR8-b.

The observed increase in the HAY could be due to an increase in the %-HA content and/or an increase in the TPY caused by increasing the virus particle number. In our study, the former possibility could be rejected. Therefore, to investigate the latter possibility, we first indirectly estimated the number of viral particles from the virus genome copy numbers in the samples. For this purpose, we quantified the HA vRNA of the mutant viruses by a real-time PCR assay. The results showed that the genome copy numbers of high-TPY and high-HAY viruses were higher than those of low-TPY and low-HAY viruses. We further examined whether the TPY and HAY are associated with the numbers of viral particles by SEM. The results showed that the virus with a high TPY presented higher viral particle numbers than the virus with a low TPY. Taken together, these results suggested that an increase in the viral particle numbers implies an increase in the TPY rather than the %-HA content, leading to a greater HAY. Although a larger viral particle size could increase the amount of total protein and HA antigens on a particle, the results for G-CVV and G/PR8-b in this study were not sufficient to demonstrate the relationship between viral particle size and TPY and HAY.

In our study, no significant increase in the HA titers and EID_50_ titers was observed for the viruses with improved HAYs. Although we did not examine the viral particle numbers needed for the hemagglutination ability and infectivity of each virus, it is likely that no increase was observed because the increase in particle numbers was not sufficiently large to affect the biological activity of the improved viruses.

Previously, Harvey and colleagues [3, 23] found a significant improvement in the HAYs of the H1N1pdm09 and H5N1 CVVs based on improved virus growth in eggs, rather than an increase in the %-HA content. However, these researchers did not determine the underlying mechanism. The results of our study implied that the HAYs of low-HAY viruses could be increased by improving virus growth, rather than increasing the %-HA content. We further inferred that the improvement in virus growth increased the number of viral particles, which led to increases in the TPY and HAY.

Although our improved mutant viruses exhibited only an approximately twofold increase in the HAY compared with that of the original CVVs, given the scale of vaccine production in eggs, a doubling of the HAY could reduce the time and number of eggs needed to produce a given amount of vaccines; for example, the number of eggs used could be reduced from 2 million to 1 million, which is a difference worth considering.

In conclusion, we revealed that high-HAY viruses produce a large number of viral particles during a given growth period and consequently exhibit a high TPY, suggesting that the TPY value could be one of the quantitative indicators to estimate the HAY level. We also showed that modification of the 3’NCR/HA sequence or replacing the master virus effectively improved the HAY. If a CVV shows a TPY above a certain level, increasing the %-HA content may be one solution to further improve the virus HAY. However, if the TPY of a CVV is low, it would be more effective to improve the TPY, (i.e., to increase the number of viral particles).

## Supporting information

S1 Fig SDS‒PAGE analysis of viral concentrates of 26 CVVs. The SDS‒PAGE analysis was performed as described in the text and the legend for Fig 2. In the results for the J-, K-, L-, and T-CVVs, a closed triangle and an open triangle represent HA2 and HA0, respectively.
(PDF)

S2 Fig NCR sequences of each CVV. Terminal nucleotide sequences of the 3’ and 5’ ends of the HA and NA vRNAs (negative sense) of each CVV are shown. Nucleotide numbering starts from the 1^st^ nucleotide of the 3’NCR. The coding regions of each CVV are indicated by CR.
(PDF)

S3 Fig Number and distribution of viral particles. Total number and distribution (%) of viral particles within the specified particle size range in the 20 fields of view. The protein concentration of each sample was 40 μg/ml.
(PDF)

S1 Table Candidate vaccine viruses (CVVs) of H5N1 and H5NX subtypes. (XLSX)

S2 Table List of primers used in this study. (XLSX)

S3 Table Differences in the amino acid sequences of the viral proteins encoded by internal genes of PR8-a and PR8-b. (XLSX)

S4 Table Characterization of each CVV. (XLSX)

S5 Table Genetic information of 3’NCR/HA mutants. (XLSX)

S6 Table Characterization of 3’NCR/HA mutants and of master virus mutants. (XLSX)

S7 Table Differences in the amino acid sequences of HA and NA. (XLSX)

S1 Raw images (PDF)

## References

[1] Johnson A, Chen LM, Winne E, Santana W, Metcalfe MG, Mateu-Petit G, et al. Identification of Influenza A/PR/8/34 Donor Viruses Imparting High Hemagglutinin Yields to Candidate Vaccine Viruses in Eggs. PLOS One. 2015;10: e0128982. doi: 10.1371/journal.pone.0128982 26068666 PMC4465931

[2] Horimoto T, Murakami S, Muramoto Y, Yamada S, Fujii K, Kiso M, et al. Enhanced growth of seed viruses for H5N1 influenza vaccines. Virology. 2007;366: 23–27. doi: 10.1016/j.virol.2007.07.002 17651774 PMC2827194

[3] Harvey R, Nicolson C, Johnson RE, Guilfoyle KA, Major DL, Robertson JS, et al. Improved haemagglutinin antigen content in H5N1 candidate vaccine viruses with chimeric haemagglutinin molecules. Vaccine. 2010;28: 8008–8014. doi: 10.1016/j.vaccine.2010.09.006 20934460

[4] Abt M, de Jonge J, Laue M, Wolff T. Improvement of H5N1 influenza vaccine viruses: influence of internal gene segments of avian and human origin on production and hemagglutinin content. Vaccine 2011;29: 5153–5162. doi: 10.1016/j.vaccine.2011.05.036 21624413

[5] Pan W, Dong Z, Meng W, Zhang W, Li T, Li C, et al. Improvement of influenza vaccine strain A/Vietnam/1194/2004 (H5N1) growth with the neuraminidase packaging sequence from A/Puerto Rico/8/34. Hum Vaccines Immunother. 2012;8: 252–259. doi: 10.4161/hv.18468 22426370

[6] Jing X, Soto J, Gao Y, Phy K, Ye Z. Assessment of viral replication in eggs and HA protein yield of pre-pandemic H5N1 candidate vaccine viruses. Vaccine. 2013;31: 4091–4097. doi: 10.1016/j.vaccine.2013.07.011 23867014

[7] Johnson RE, Hamill M, Harvey R, Nicolson C, Robertson JS, Engelhardt OG. Permissible Variation in the 3′ Non-Coding Region of the Haemagglutinin Genome Segment of the H5N1 Candidate Influenza Vaccine Cirus NIBRG-14. PLOS ONE. 2012;7: e36241. doi: 10.1371/journal.pone.0036241 22606247 PMC3350513

[8] Jing X, Phy K, Li X, Ye Z. Increased hemagglutinin content in a reassortant 2009 pandemic H1N1 influenza virus with chimeric neuraminidase containing donor A/Puerto Rico/8/34 virus transmembrane and stalk domains. Vaccine. 2012;30: 4144–4152. doi: 10.1016/j.vaccine.2012.04.073 22561313

[9] Shirakura M, Kawaguchi A, Tashiro M, Nobusawa E. Composition of Hemagglutinin and Neuraminidase Affects the Antigen Yield of Influenza A(H1N1)pdm09 Candidate Vaccine Viruses. Jpn J Infect Dis. 2013;66: 65–68. doi: 10.7883/yoken.66.65 23429089

[10] Harvey R, Hamill M, Robertson JS, Minor PD, Vodeiko GM, Weir JP, et al. Application of deglycosylation to SDS PAGE analysis improves calibration of influenza antigen standards. Biologicals. 2012;40: 96–99. doi: 10.1016/j.biologicals.2011.12.009 22244521

[11] Nakamura K, Shirakura M, Suzuki Y, Naito T, Fujisaki S, Tashiro M, et al. Development of a high-yield reassortant influenza vaccine virus derived from the A/Anhui/1/2013 (H7N9) strain. Vaccine. 2016;34: 328–333. doi: 10.1016/j.vaccine.2015.11.050 26657023

[12] Shevchenko A, Wilm M, Vorm O, Mann M. Mass Spectrometric Sequencing of Proteins from Silver-Stained Polyacrylamide Gels. Anal Chem. 1996;68: 850–858. doi: 10.1021/ac950914h 8779443

[13] R Core Team. R: The R Project for Statistical Computing. In: R: A language and environment for statistical computing. R Foundation for Statistical Computing, Vienna, Austria. [Internet]. 2021 [cited 3 Oct 2022]. Available: https://www.r-project.org/

[14] Neumann G, Watanabe T, Ito H, Watanabe S, Goto H, Gao P, et al. Generation of influenza A viruses entirely from cloned cDNAs. Proc Natl Acad Sci. 1999;96: 9345–9350. doi: 10.1073/pnas.96.16.9345 10430945 PMC17785

[15] Dong J, Matsuoka Y, Maines TR, Swayne DE, O’Neill E, Davis CT, et al. Development of a new candidate H5N1 avian influenza virus for pre-pandemic vaccine production. Other Respir Viruses. 2009;3: 287–295. doi: 10.1111/j.1750-2659.2009.00104.x 19903211 PMC4941393

[16] Li C, Shao M, Cui X, Song Y, Li J, Yuan L, et al. Application of deglycosylation and electrophoresis to the quantification of influenza viral hemagglutinins facilitating the production of 2009 pandemic influenza (H1N1) vaccines at multiple manufacturing sites in China. Biologicals. 2010;38: 284–289. doi: 10.1016/j.biologicals.2009.12.004 20074976

[17] Tretter V, Altmann F, März L. Peptide-N4-(N-acetyl-beta-glucosaminyl) asparagine amidase F cannot release glycans with fucose attached α1 → 3 to the asparagine-linked N-acetylglucosamine residue. Eur J Biochem. 1991;199: 647–652. doi: 10.1111/j.1432-1033.1991.tb16166.x 1868849

[18] Harvey R., Wheeler J.X., Wallis C.L., Robertson J.S. and Engelhardt O.G. Quantitation of haemagglutinin H5N1 influenza viruses reveals low haemagglutinin content of vaccine virus NIBRG-14 (H5N1). Vaccine. 2008;26: 6550–6554. doi: 10.1016/j.vaccine.2008.09.050 18840494

[19] Robertson JS, Nicolson C, Harvey R, Johnson R, Major D, Guilfoyle K, et al. The development of vaccine viruses against pandemic A(H1N1) influenza. Vaccine. 2011;29: 1836–1843. doi: 10.1016/j.vaccine.2010.12.044 21199698

[20] Harvey R, Guilfoyle KA, Roseby S, Robertson JS, Engelhardt OG. Improved Antigen Yield in Pandemic H1N1 (2009) Candidate Vaccine Viruses with Chimeric Hemagglutinin Molecules. J Virol. 2011;85: 6086–6090. doi: 10.1128/JVI.00096-11 21490098 PMC3126323

[21] Zhao L, Peng Y, Zhou K, Cao M, Wang J, Wang X, et al. New Insights into the Nonconserved Noncoding Region of the Subtype-Determinant Hemagglutinin and Neuraminidase Segments of Influenza A Viruses. J Virol. 2014;88: 11493–11503. doi: 10.1128/JVI.01337-14 25056889 PMC4178829

[22] Wang J, Li J, Zhao L, Cao M, Deng T. Dual Roles of the Hemagglutinin Segment-Specific Noncoding Nucleotides in the Extended Duplex Region of the Influenza A Virus RNA Promoter. J Virol. 2016;91: e01931–16. doi: 10.1128/JVI.01931-16 27795444 PMC5165193

[23] Xiao Y, Zhang W, Pan M, Bauer DLV, Bi Y, Cao M, et al. Synergistic Effect between 3’-Terminal Noncoding and Adjacent Coding Regions of the Influenza A Virus Hemagglutinin Segment on Template Preference. J Virol. 2021;95: e00878–21. doi: 10.1128/JVI.00878-21 34190596 PMC8387048

[24] Lee I, Il Kim J, Park S, Bae JY, Yoo K, Yun S-H, et al. Single PA mutation as a high yield determinant of avian influenza vaccines. Sci Rep. 2017;7: 40675. doi: 10.1038/srep40675 28084423 PMC5233958

[25] Plant EP, Ye Z. Chimeric neuraminidase and mutant PB1 gene constellation improves growth and yield of H5N1 vaccine candidate virus. J Gen Virol. 2015;96: 752–755. doi: 10.1099/jgv.0.000025 25502649

