# Supplementary material for: Identification of the properties of H5 influenza vaccine viruses with high hemagglutinin yields
Source: PLoS One. 2023 Jan 20;18(1):e0280811. doi: 10.1371/journal.pone.0280811 (PMC9858889; doi:10.1371/journal.pone.0280811)
Supplement: S1 Fig — The SDS‒PAGE analysis was performed as described in the text and the legend for Fig 2. In the results for the J-, K-, L-, and T-CVVs, a closed triangle and an open triangle represent HA2 and HA0, respectively. (PDF) [file pone.0280811.s001.pdf]

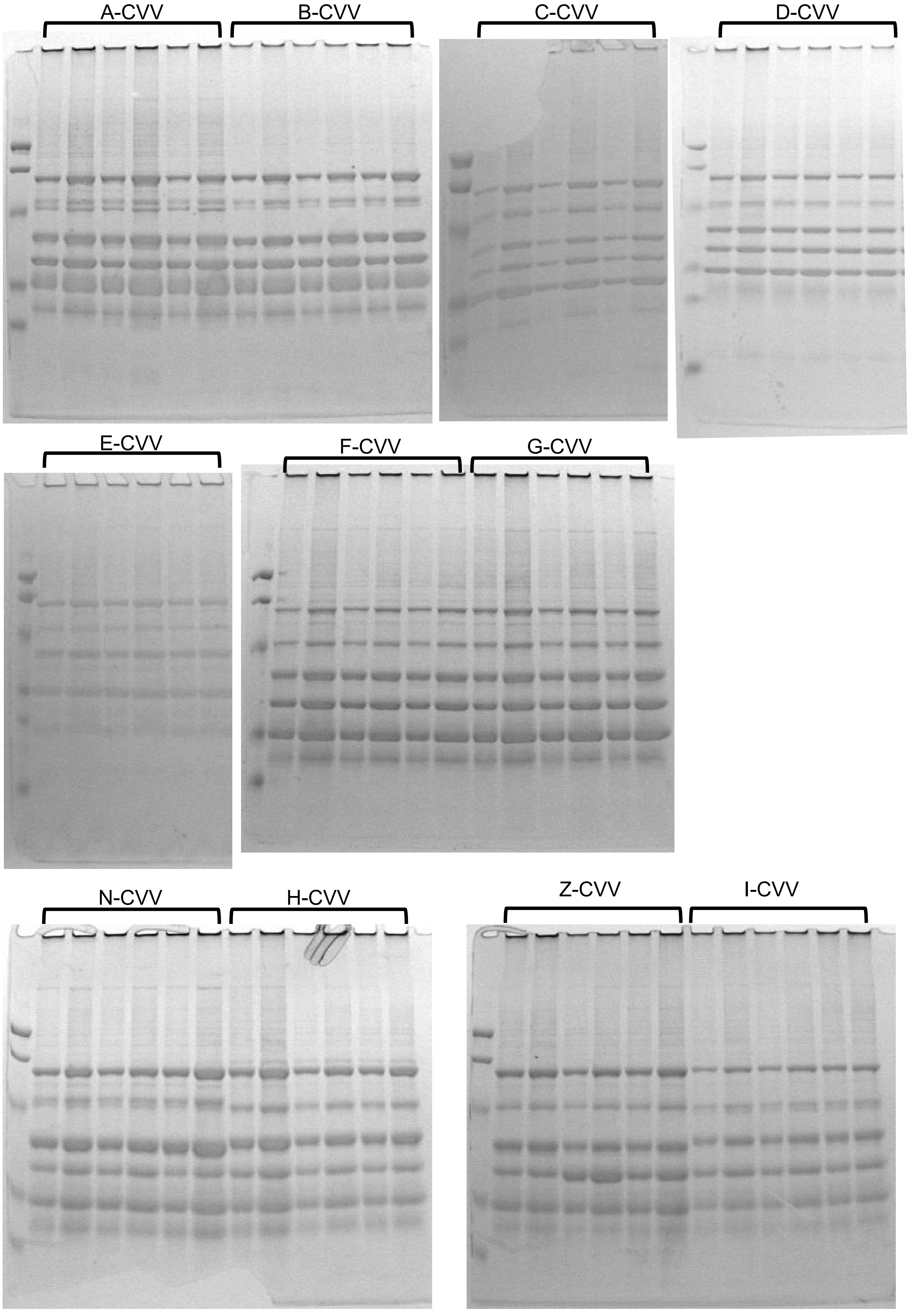
A-CVV
B-CVV
C-CVV
D-CVV
E-CVV
F-CVV
G-CVV
N-CVV
H-CVV
Z-CVV
I-CVV

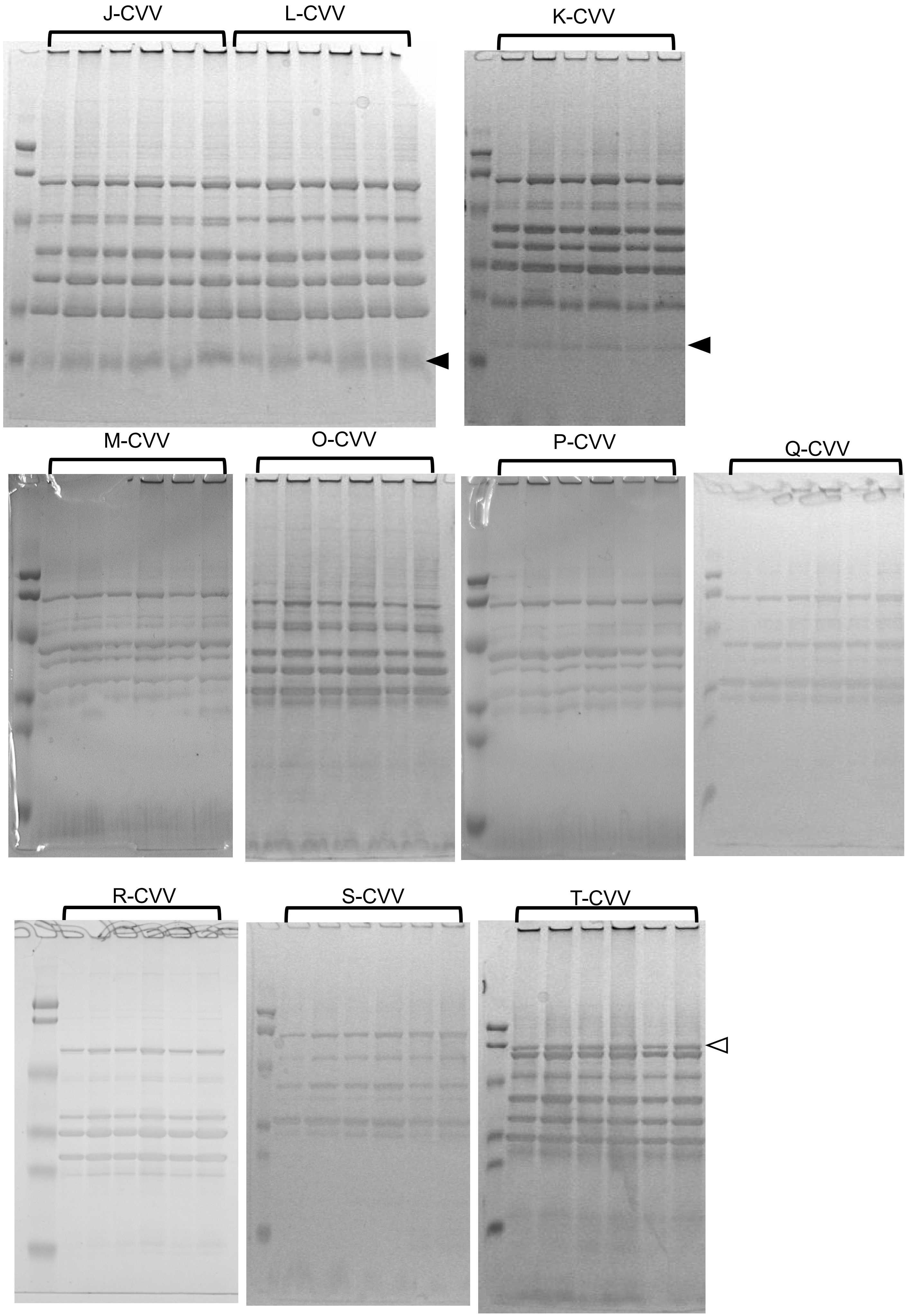
J-CVV
L-CVV
K-CVV
M-CVV
O-CVV
P-CVV
Q-CVV
R-CVV
S-CVV
T-CVV

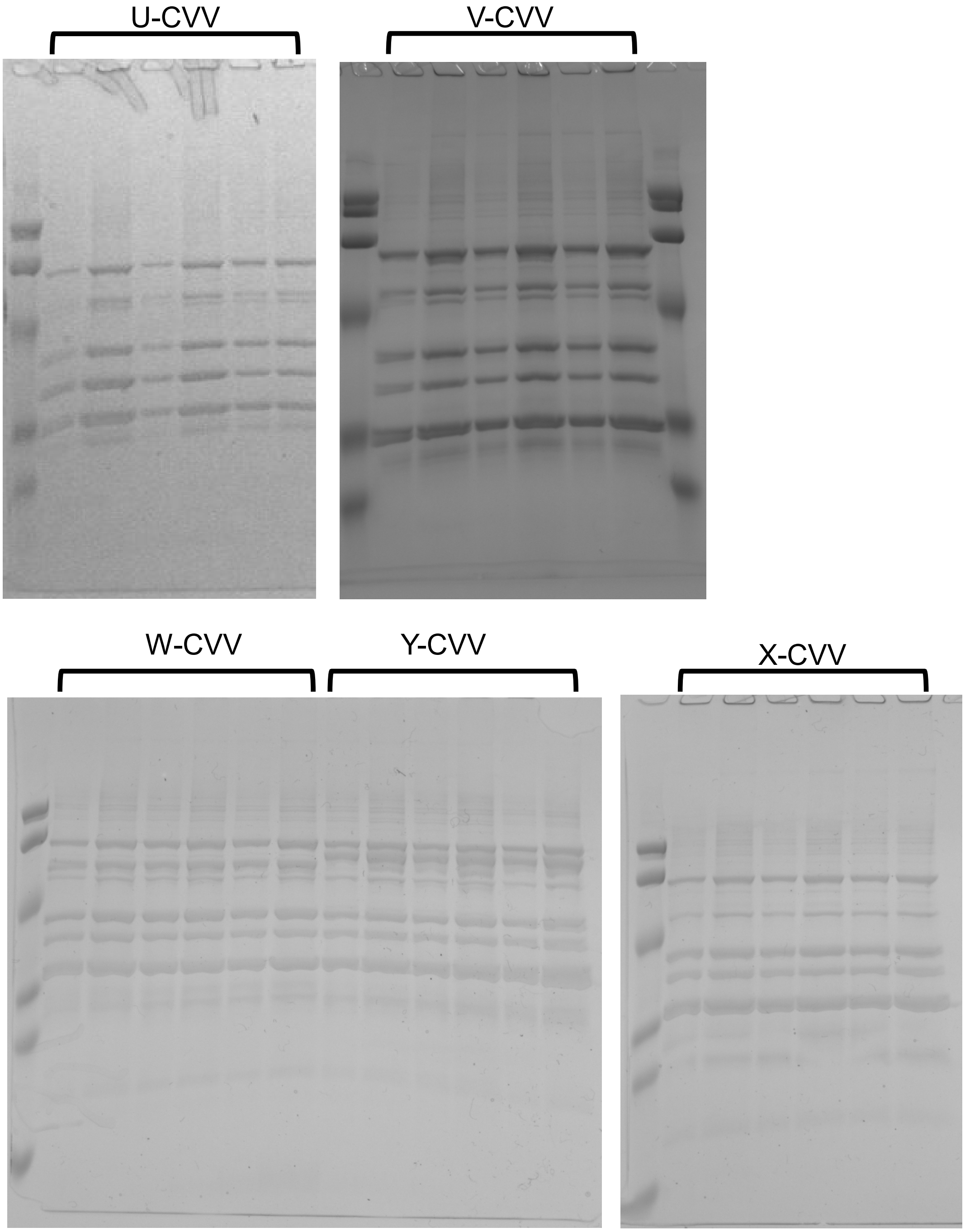
U-CVV
V-CVV
W-CVV
Y-CVV
X-CVV
