# Supplementary figures and images for: Identification of the properties of H5 influenza vaccine viruses with high hemagglutinin yields
Source: PLoS One. 2023 Jan 20;18(1):e0280811. doi: 10.1371/journal.pone.0280811 (PMC9858889; doi:10.1371/journal.pone.0280811)

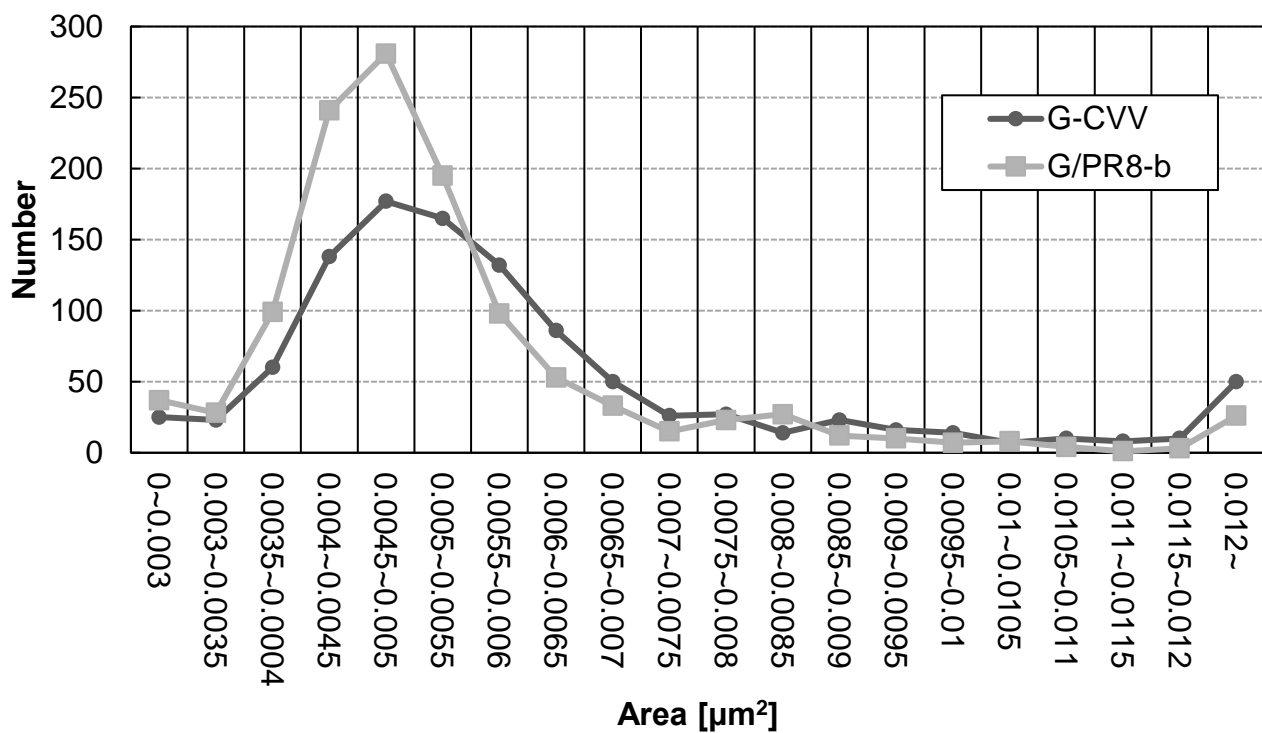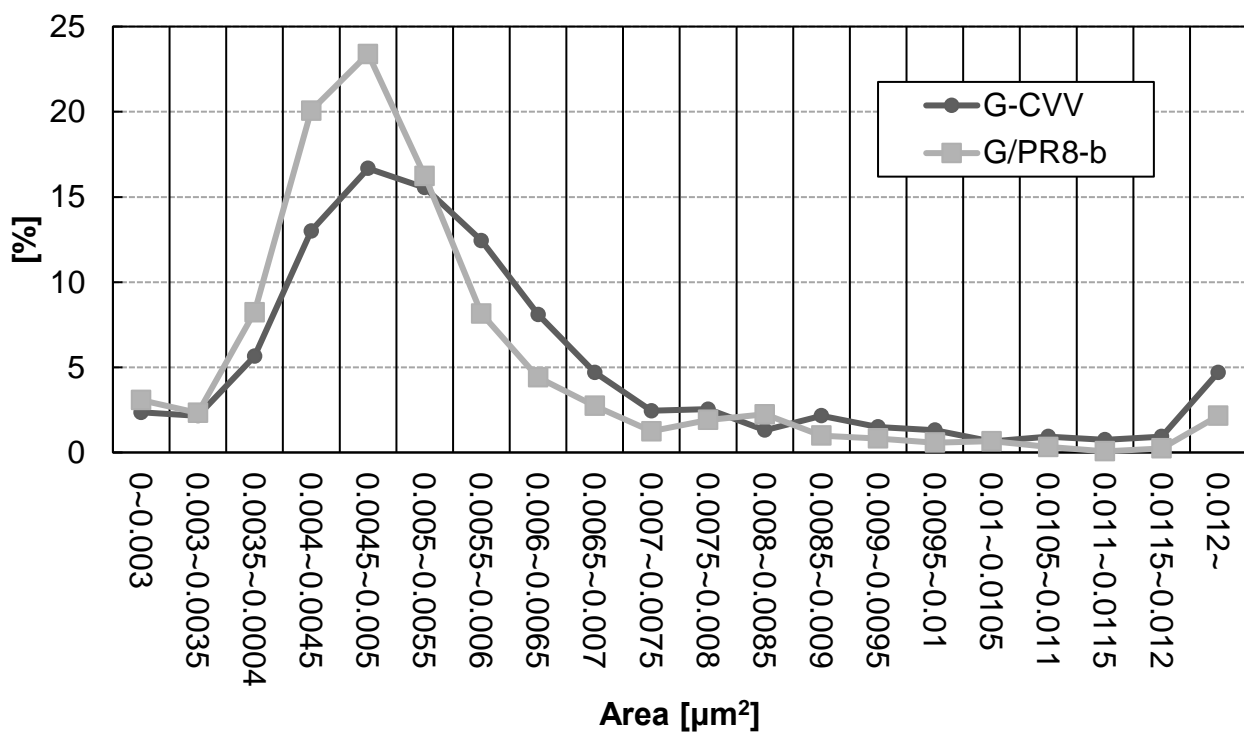

Supplement: S3 Fig — Total number and distribution (%) of viral particles within the specified particle size range in the 20 fields of view. The protein concentration of each sample was 40 μg/ml. (PDF) [file pone.0280811.s003.pdf]
