# Supplementary material for: Identification of the properties of H5 influenza vaccine viruses with high hemagglutinin yields
Source: PLoS One. 2023 Jan 20;18(1):e0280811. doi: 10.1371/journal.pone.0280811 (PMC9858889; doi:10.1371/journal.pone.0280811)
Supplement: S1 Raw images — (PDF) [file pone.0280811.s011.pdf]

Figure 2

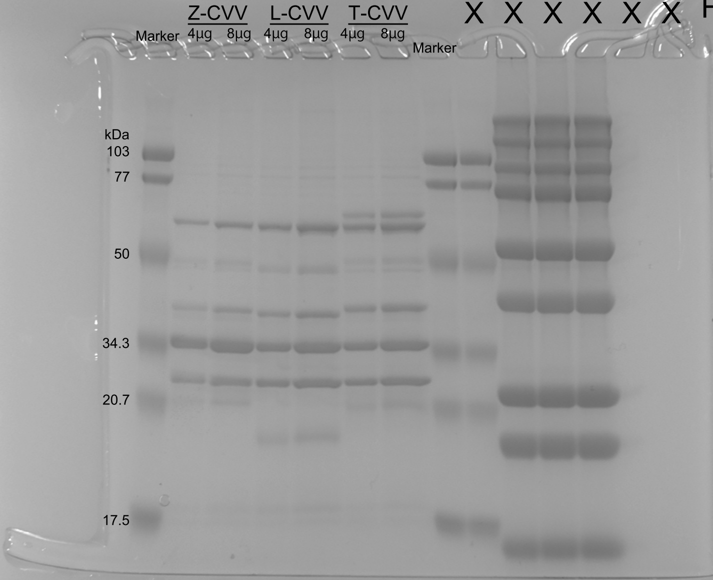

Automatically captured by the LuminoGraph I (ATTO Corporation, Tokyo, Japan)

S1 Fig

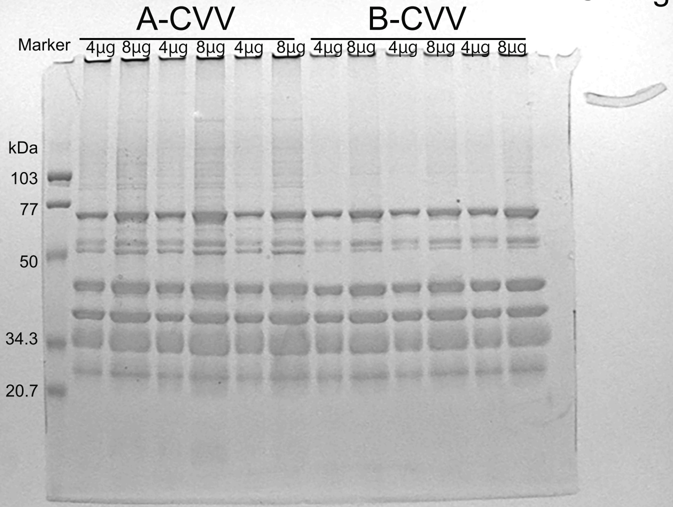

Automatically captured by the LuminoGraph I (ATTO Corporation, Tokyo, Japan)

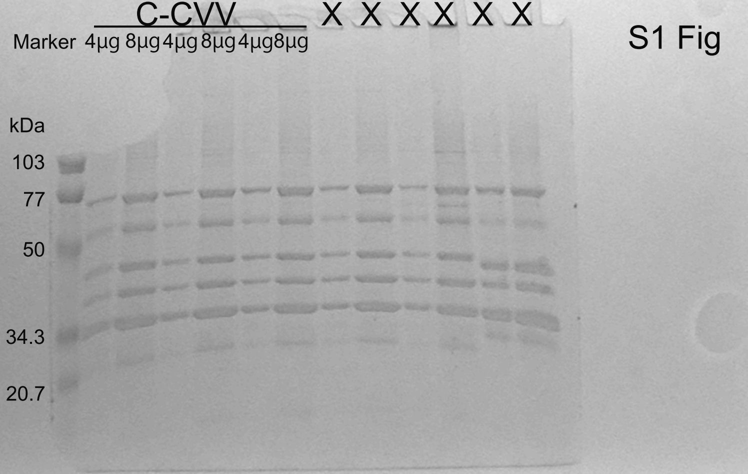

Automatically captured by the LuminoGraph I (ATTO Corporation, Tokyo, Japan)

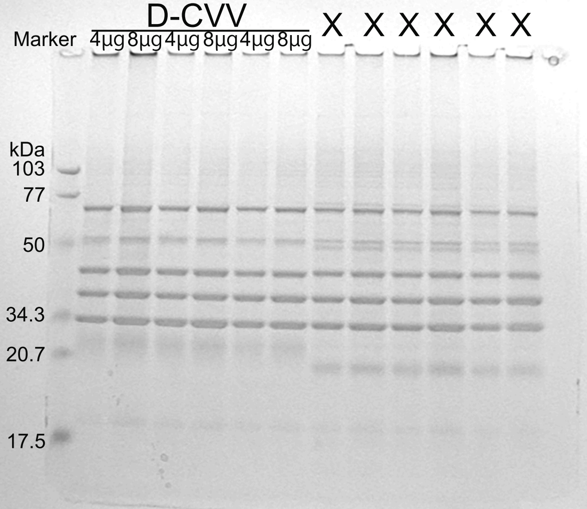

Automatically captured by the LuminoGraph I (ATTO Corporation, Tokyo, Japan)

S1 Fig

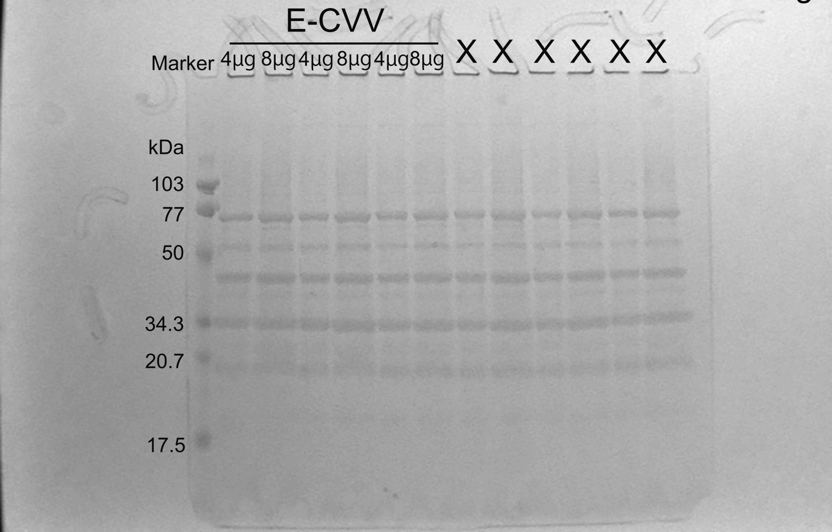

Automatically captured by the LuminoGraph I (ATTO Corporation, Tokyo, Japan)

S1 Fig

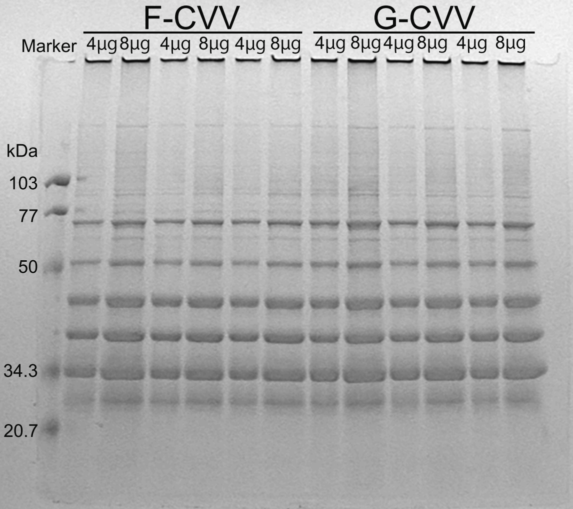

Automatically captured by the LuminoGraph I (ATTO Corporation, Tokyo, Japan)

S1 Fig

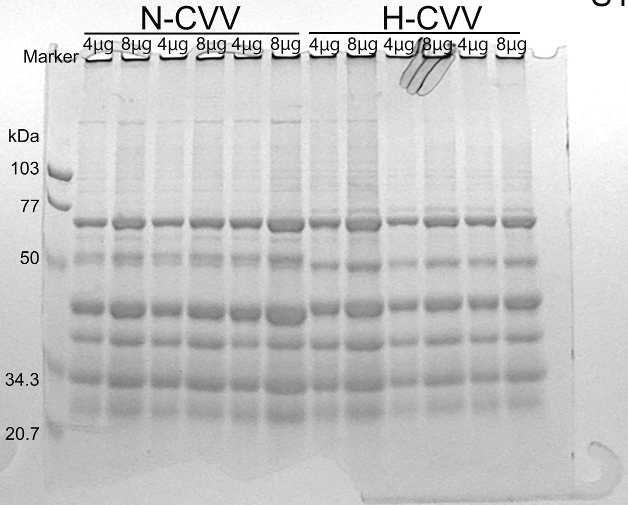

Automatically captured by the LuminoGraph I (ATTO Corporation, Tokyo, Japan)

S1 Fig

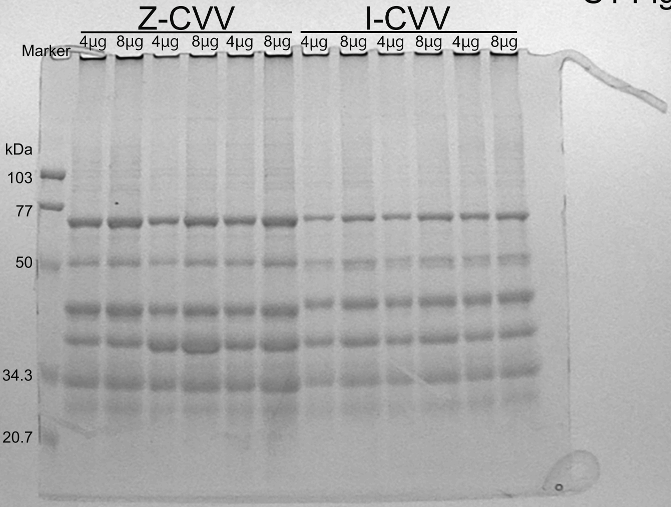

Automatically captured by the LuminoGraph I (ATTO Corporation, Tokyo, Japan)

S1 Fig

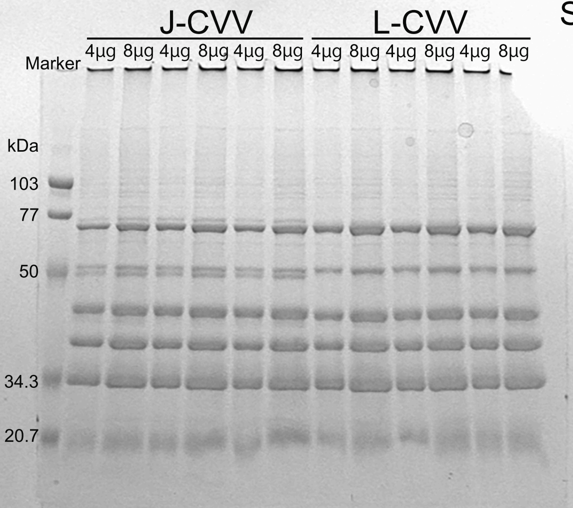

Automatically captured by the LuminoGraph I (ATTO Corporation, Tokyo, Japan)

S1 Fig

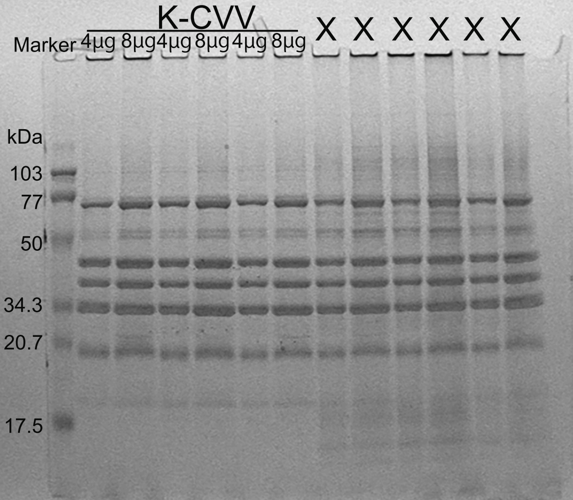

Automatically captured by the LuminoGraph I (ATTO Corporation, Tokyo, Japan)

S1 Fig

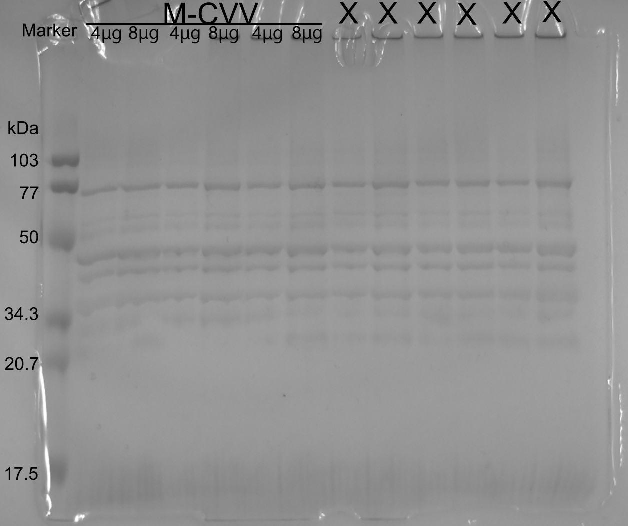

Automatically captured by the LuminoGraph I (ATTO Corporation, Tokyo, Japan)

S1 Fig

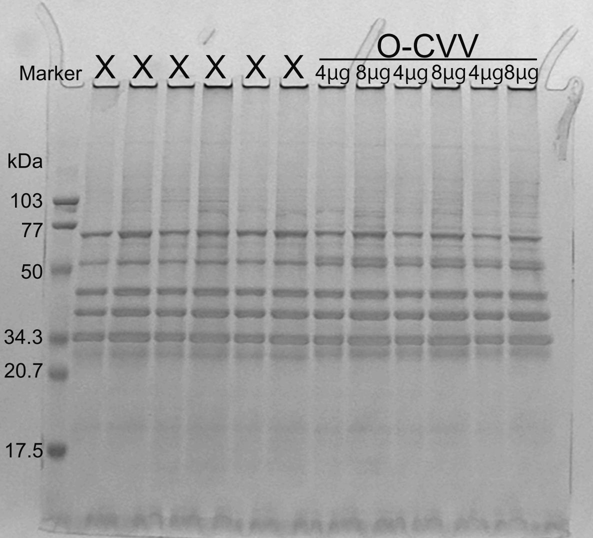

Automatically captured by the LuminoGraph I (ATTO Corporation, Tokyo, Japan)

S1 Fig

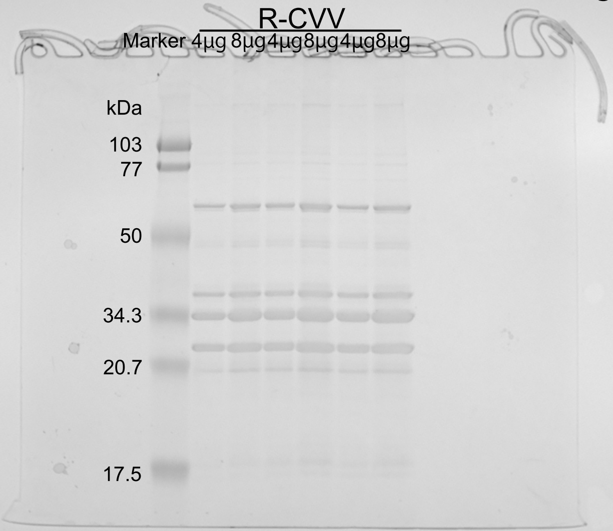

Automatically captured by the LuminoGraph I (ATTO Corporation, Tokyo, Japan)

S1 Fig

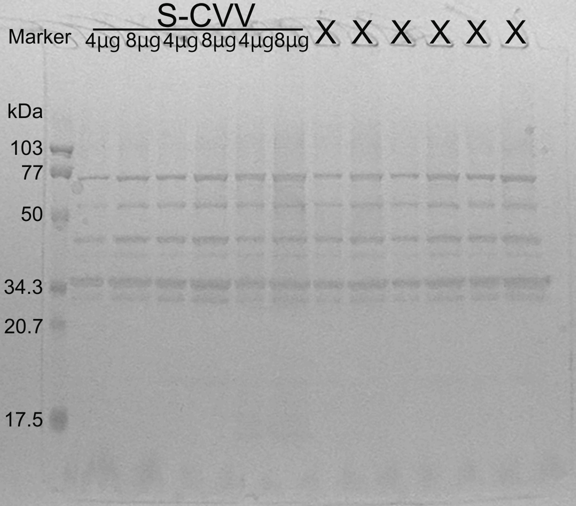

Automatically captured by the LuminoGraph I (ATTO Corporation, Tokyo, Japan)

S1 Fig

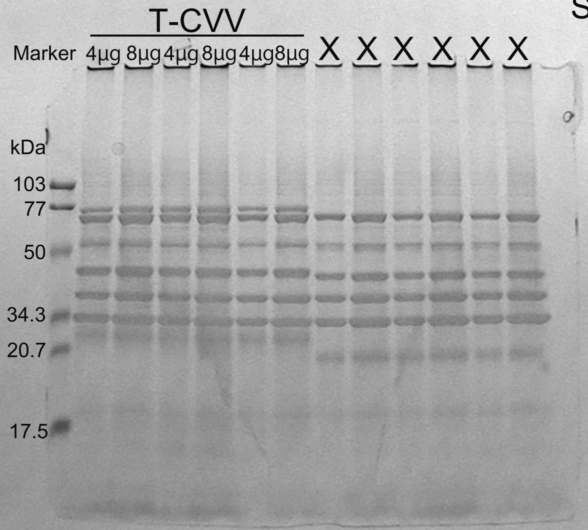

Automatically captured by the LuminoGraph I (ATTO Corporation, Tokyo, Japan)

S1 Fig

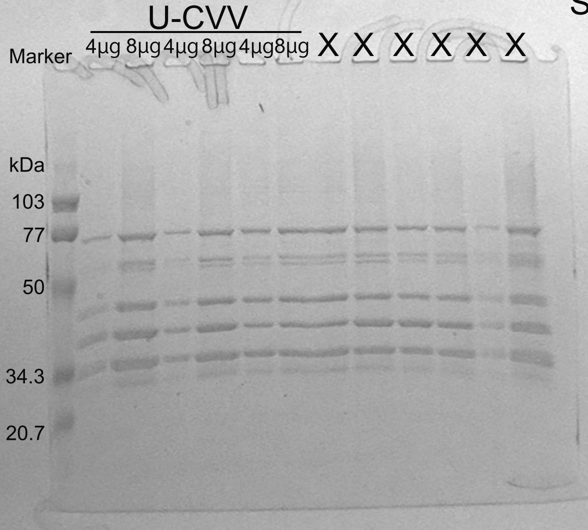

Automatically captured by the LuminoGraph I (ATTO Corporation, Tokyo, Japan)

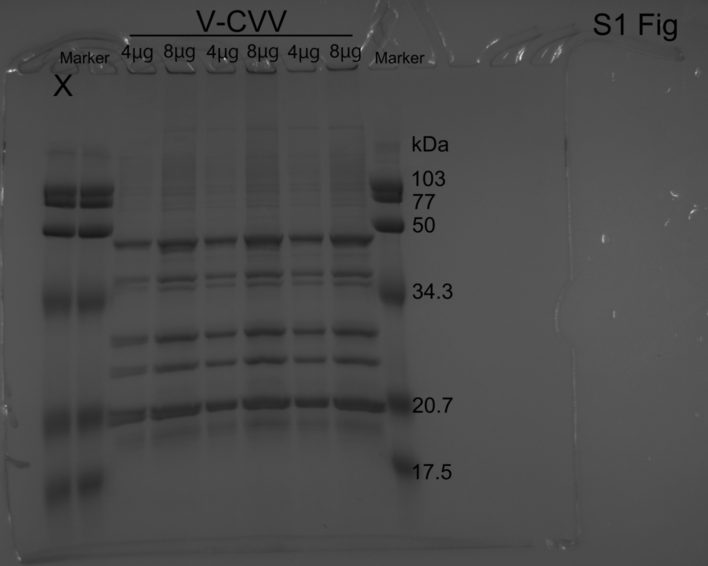

Automatically captured by the LuminoGraph I (ATTO Corporation, Tokyo, Japan)

S1 Fig

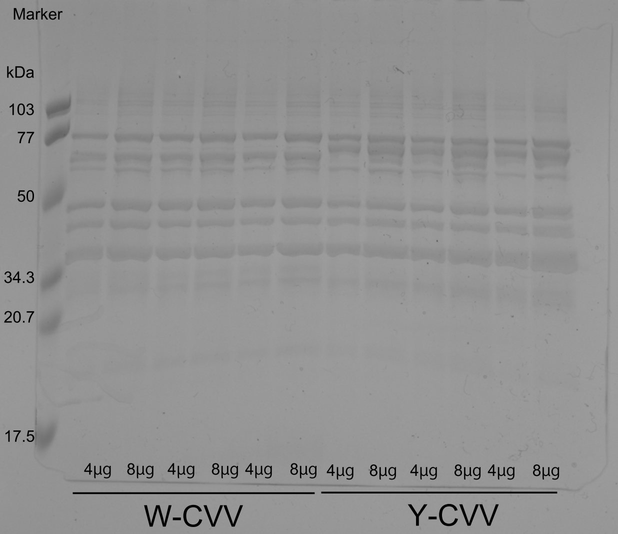

Automatically captured by the LuminoGraph I (ATTO Corporation, Tokyo, Japan)

S1 Fig

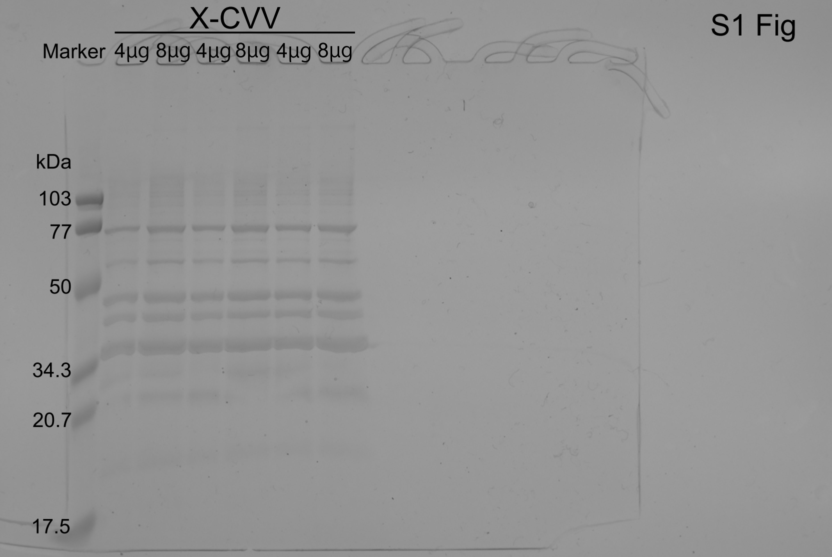

Automatically captured by the LuminoGraph I (ATTO Corporation, Tokyo, Japan)
