# Supplementary material for: Identification of the properties of H5 influenza vaccine viruses with high hemagglutinin yields
Source: PLoS One. 2023 Jan 20;18(1):e0280811. doi: 10.1371/journal.pone.0280811 (PMC9858889; doi:10.1371/journal.pone.0280811)
Supplement: S2 Fig — Terminal nucleotide sequences of the 3’ and 5’ ends of the HA and NA vRNAs (negative sense) of each CVV are shown. Nucleotide numbering starts from the 1st nucleotide of the 3’NCR. The coding regions of each CVV are indicated by CR. (PDF) [file pone.0280811.s002.pdf]

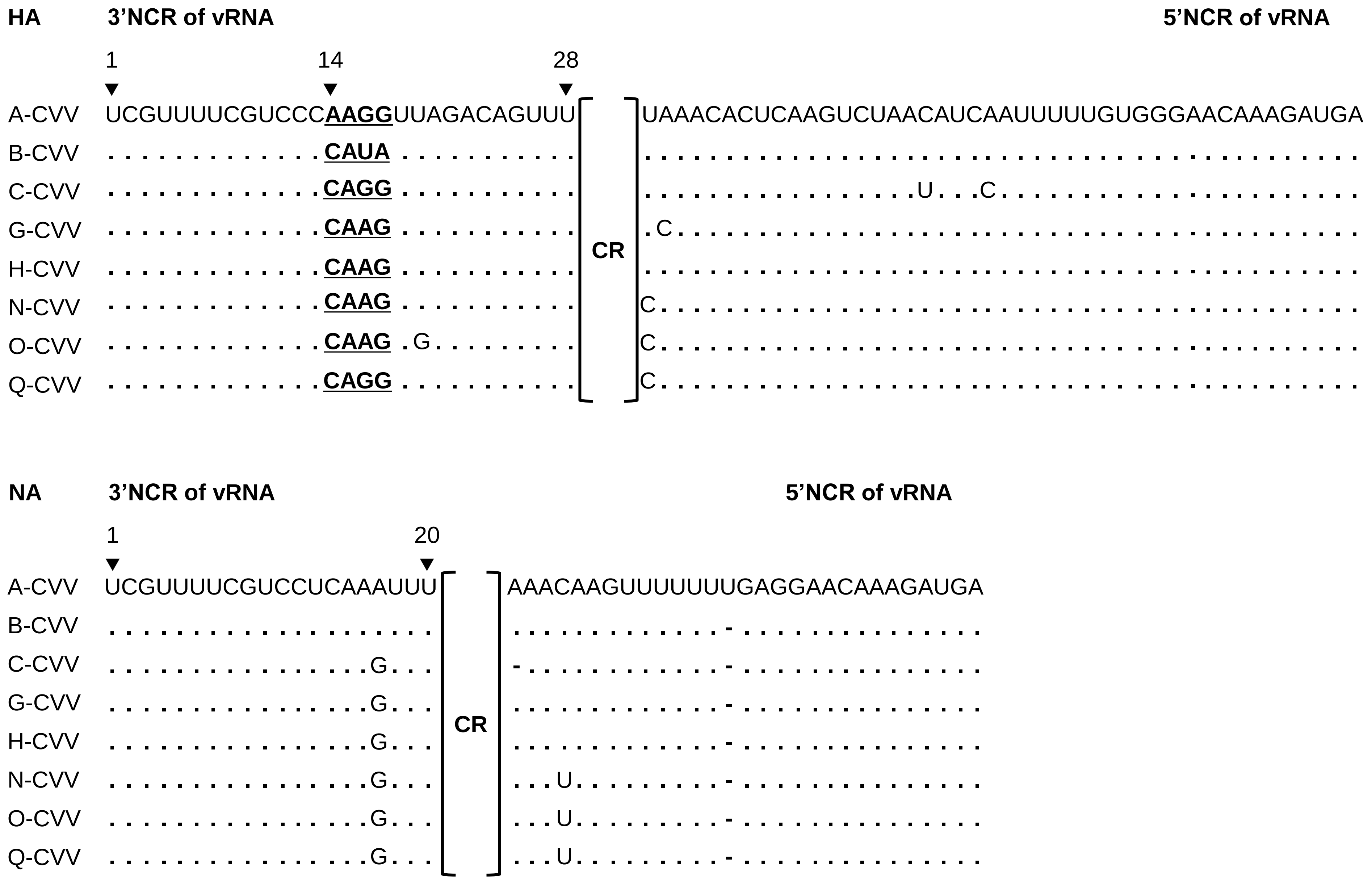
HA
3’NCR of vRNA
5’NCR of vRNA
1
14
28
A-CVV
UCGUUUUCGUCCCAAGGUUAGACAGUUU
UAAACACUCAAGUCUAACAUCAAUUUUUGUGGGAACAAAGAUGA
B-CVV
CAUA
C-CVV
CAGG
U
C
G-CVV
CAAG
C
H-CVV
CAAG
N-CVV
CAAG
C
O-CVV
CAAG
G
C
Q-CVV
CAGG
C
CR
NA
3’NCR of vRNA
5’NCR of vRNA
1
20
A-CVV
UCGUUUUCGUCCUCAAAUUU
AAACAAGUUUUUUUGAGGAACAAAGAUGA
B-CVV
C-CVV
G
G-CVV
G
H-CVV
G
N-CVV
G
U
O-CVV
G
U
Q-CVV
G
U
CR
